# The spleen tyrosine kinase inhibitor entospletinib resolves inflammation to promote repair following acute kidney injury

**DOI:** 10.1172/jci.insight.189601

**Published:** 2025-08-22

**Authors:** Esteban E. Elias, Arthur Lau, Sisay Getie Belay, Afshin Derakhshani, Graciela Andonegui, Craig N. Jenne, Antoine Dufour, Nathan A. Bracey, Justin Chun, Daniel A. Muruve

**Affiliations:** 1 Department of Medicine,; 2 Department of Physiology and Pharmacology, and; 3 Department of Microbiology, Immunology and Infectious Diseases, Snyder Institute for Chronic Diseases, University of Calgary, Alberta, Canada.

**Keywords:** Inflammation, Nephrology, Chronic kidney disease, Drug therapy, Macrophages

## Abstract

Nonresolving inflammation and maladaptive renal repair contribute to the pathogenesis of acute kidney injury (AKI) transition to chronic kidney disease (CKD). Few therapies have been identified that can modulate these injurious pathways following AKI. Spleen tyrosine kinase (SYK) is an immune regulator expressed in the kidney and a potential therapeutic target for AKI. The effect of the selective SYK inhibitor entospletinib was studied in AKI-to-CKD transition. Entospletinib was administered to mice undergoing unilateral renal ischemia-reperfusion injury (IRI), with kidneys analyzed over 14 days. Single-cell RNA sequencing, digital spatial profiling, intravital microscopy, and flow cytometry were employed to study renal phenotypes. Entospletinib administered before and after IRI protected ischemic kidneys and significantly attenuated the transition to CKD. Entospletinib targeted leukocyte-expressed SYK and prevented neutrophil/monocyte recruitment to the kidney. Entospletinib reduced nonresolving tubulointerstitial inflammation after AKI by blocking activation of mannose receptor-1– and C-type lectin domain family 7 member A–expressing proinflammatory macrophages. The resolution of renal inflammation mediated by entospletinib was associated with a reciprocal increase in resident macrophages, reparative gene expression, preserved tubular integrity, and reduced renal fibrosis. The SYK inhibitor entospletinib resolves renal inflammation and promotes repair following AKI.

## Abstract 2

The SYK inhibitor, entospletinib prevents non-resolving renal inflammation and enhances repair pathways in the kidney following acute injury limiting the development of chronic kidney disease.

## Graphical abstract

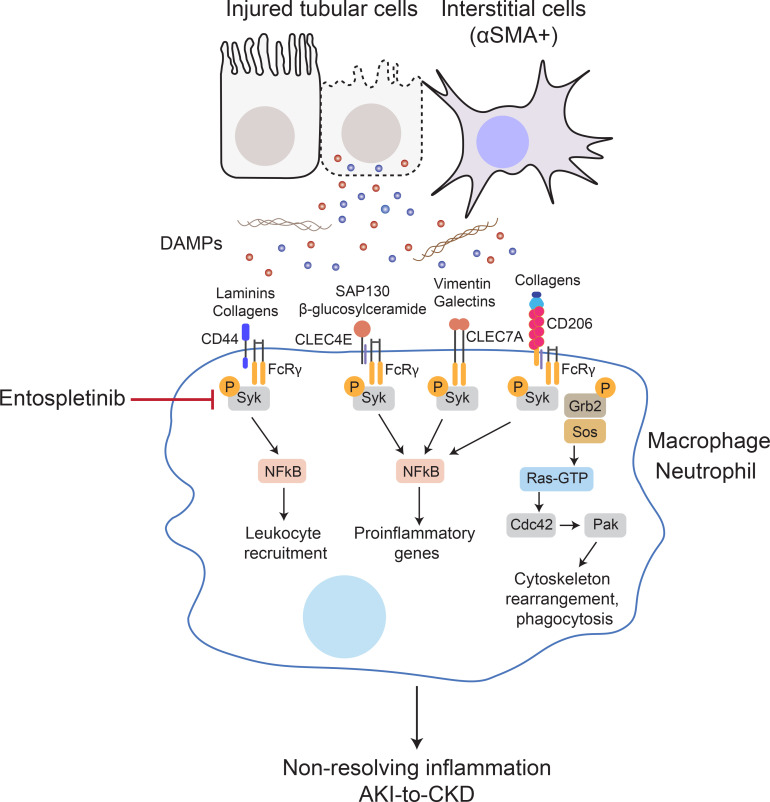

## Introduction

Acute kidney injury (AKI) is associated with significant morbidity and mortality. Aside from supportive care, currently there are no specific therapies available for AKI (1, 2). Patients with AKI from a variety of causes such as sepsis or ischemia-reperfusion injury (IRI) are at increased risk of progression to chronic kidney disease (CKD), a process termed AKI-to-CKD transition (3). Tubular injury and persistent or nonresolving renal inflammation that follow severe AKI results in maladaptive repair, loss of functional tissue, and excessive ECM deposition that leads to CKD (4–7). The development of CKD after AKI further increases the risk of adverse patient outcomes, such as end-stage kidney disease, cardiovascular disease, and death (8, 9).

Kidney repair is a complex biological process that is activated during injury to resolve inflammation, maintain cellular integrity, and enhance cellular regeneration to preserve kidney structure and function. Virtually all cell types in the kidney participate in repair, including tubular epithelium, endothelium, stroma, and leukocytes (10–14). While inflammation contributes to kidney injury, leukocytes, in particular macrophages, are essential to promote renal repair (12, 13, 15). Both resident and recruited renal macrophages display plasticity during injury and can switch from a proinflammatory to an antiinflammatory and reparative phenotype based on signals derived within the injured renal microenvironment. We and others have characterized many of the factors that regulate this phenotypic switch, including cytokines, growth factors, and danger-associated molecular patterns (DAMPs) released from injured tubular cells, perivascular and other interstitial stromal cells, as well as macrophages themselves (4–6, 10, 16–23). Despite these studies, it is evident that kidney macrophages exist on a spectrum with overlapping cell surface markers and functions (15), decreasing the likelihood that therapeutic targeting of distinct macrophage populations could be achieved to improve renal outcomes after AKI. Rather, altering the balance between kidney inflammation and repair may be a more effective strategy to shape the phenotype of the AKI-to-CKD transition. Potential therapies for AKI targeting injury prevention and/or early events such as acute inflammation or tubular cell death have been reported (4, 24–26); however, few clinically viable pharmacologic agents have been identified that can modulate post-AKI pathways that drive nonresolving inflammation or kidney repair.

Spleen tyrosine kinase (SYK) is a non–receptor tyrosine kinase highly expressed in hematopoietic cells and involved in various biological functions, including cellular adhesion, immune recognition, and vascular development (27). SYK exerts its actions downstream of B cell, Fcγ, and C-type lectin receptors. Several SYK inhibitors such as fostamatinib and entospletinib are under clinical development primarily for hematologic disorders and B lymphocyte malignancies, with fostamatinib being first approved for the treatment of immune thrombocytopenia (28, 29). In the kidney, SYK is expressed in resident and recruited myeloid cells such as macrophages and to a much lesser degree in parenchyma such as tubular cells, mesangial cells, and interstitial myofibroblasts (30–32). SYK has been shown to participate in the pathogenesis of nonimmune experimental renal injury. For example, following IRI in mice, SYK regulates the recruitment and activation of proinflammatory neutrophils and macrophages. Pharmacologic inhibition of SYK using compounds such as CC-0417 and BAY61-3606 diminished IRI-induced acute renal inflammation and tubular injury, with reduced leukocyte recruitment and a downregulation of proinflammatory cytokines including IL-1β, TNF-α, IL-6, and CCL2 (33). Mice with SYK-deficient myeloid cells display a similar phenotype to those treated with pharmacologic inhibitors, suggesting the SYK exerts the majority of its effects in leukocytes during kidney injury (30). Consistent with these reports, C-type lectin domain family 4 member E (CLEC4E, Mincle) signaling via SYK was shown in several studies to mediate acute inflammation during experimental AKI (19, 23, 34). Finally, SYK was found to be highly expressed in renal macrophages following unilateral ureteric obstruction in mice. SYK inhibition with CC-0417 significantly reduced inflammation and fibrosis in this model (35). Thus, the existing evidence shows that SYK participates in both renal inflammation and fibrosis, establishing it as a potential therapeutic target for both AKI and CKD.

Entospletinib is a second-generation, selective, and potent SYK inhibitor that is currently in clinical development for hematologic malignancies with a favorable short-term safety profile (36, 37). In this study, entospletinib-mediated renoprotection was used as a model to interrogate inflammation and repair pathways in mice undergoing IRI and AKI-to-CKD transition. Entospletinib significantly attenuated AKI-to-CKD transition, with its major effect in resident and recruited leukocyte populations. Importantly, entospletinib blocked proinflammatory signaling in macrophages expressing the SYK-dependent receptors macrophage mannose receptor-1 (CD206) and C-type lectin domain family 7 member A (CLEC7A) that resolved inflammation and enhanced kidney repair pathways. These data identify SYK as a mediator of nonresolving renal inflammation and entospletinib as a potential therapeutic agent to mitigate AKI-to-CKD transition in humans.

## Results

### Entospletinib protects against AKI-to-CKD transition.

To explore the impact of entospletinib on AKI-to-CKD transition, we employed a model of maladaptive repair induced by unilateral renal IRI. Mice were administered intraperitoneal injections of entospletinib (20 mg/kg) or vehicle (DMSO) 30 minutes prior to IRI of the left kidney without contralateral nephrectomy. At this dose in mice, entospletinib is approximately 10-fold lower than the human equivalent dose based on allometric scaling and reached a peak plasma concentration of 1.69 μM at 6 hours following a single intraperitoneal injection, which is near the drug EC_50_ and less likely to cause off-target effects (36) (Supplemental Figure 1, A and B; supplemental material available online with this article; https://doi.org/10.1172/jci.insight.189601DS1). Treatment continued with daily injections for the first 6 days, followed by injections every other day until 14 days (Supplemental Figure 1C). In this unilateral IRI model, survival was 100% after 14 days, with no significant changes in body weight (Supplemental Figure 1D). Periodic acid–Schiff (PAS) staining of contralateral kidneys indicated that entospletinib did not affect normal renal architecture (Supplemental Figure 1E). Furthermore, entospletinib displayed minimal impact on primary human tubular epithelial cells, with no cell death observed following 24 hours of drug exposure (1 μM) in vitro (Supplemental Figure 1, F and G). Thus, consistent with the clinical experience, short-term administration of entospletinib does not cause renal toxicity in mice.

**Figure 1:**
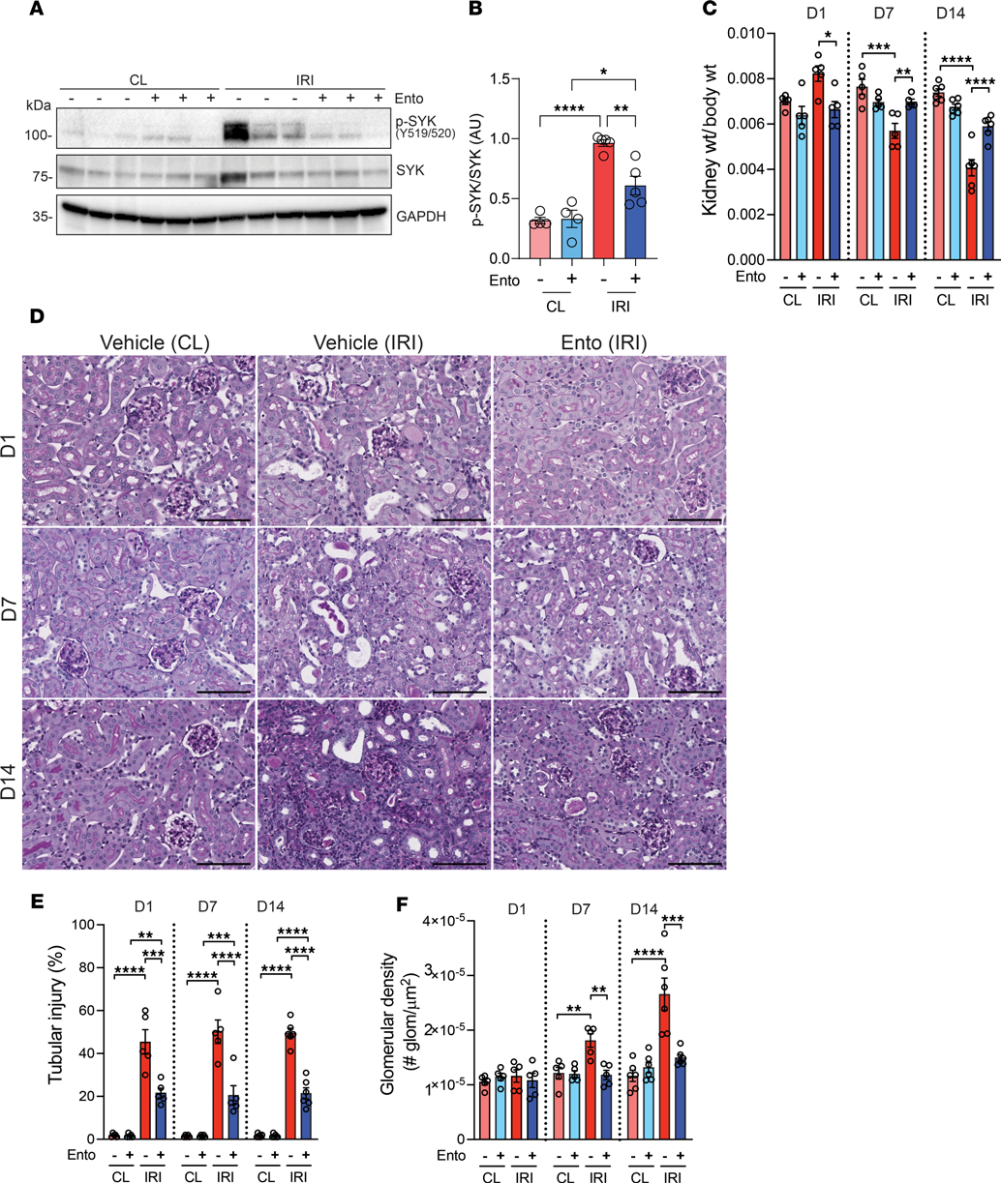
Entospletinib prevents IRI-induced AKI-to-CKD transition. Kidney pathology following ischemia reperfusion injury (IRI) on days 1, 7, and 14 in vehicle- and entospletinib-treated (Ento-treated) mice. Contralateral (CL) kidneys were used as controls. (**A**) Immunoblotting of whole kidney lysates probing for p-SYK^Y519/520^, total SYK, and GAPDH on day 1. (**B**) p-SYK quantification (densitometry mean ± SEM, *n* = 4–5). (**C**) Quantification of kidney weight/body weight ratio (mean ± SEM, *n* = 5–6). (**D**) Representative periodic acid–Schiff (PAS) kidney histopathology at the indicated treatment and time points. Scale bars: 100 μm. (**E**) Percentage of injured tubules/kidney (mean ± SEM, each data point represents average of at least 5 fields of view/kidney, *n* = 5–6). (**F**) Cortical glomerular density (mean ± SEM glomeruli/kidney, *n* = 5–6). Statistical analysis was performed using ANOVA followed by Bonferroni’s multiple-comparison test. *P < 0.05; **P < 0.01; ***P < 0.001; ****P < 0.0001.

Immunoblotting of whole kidney lysates confirmed SYK expression and phosphorylation at Y519/520 one day after IRI, which was diminished by entospletinib treatment (Figure 1, A and B), confirming that the drug effectively targeted SYK in the kidney. On days 7 and 14 after IRI, macroscopic changes were evident in ischemic kidneys from vehicle-treated mice, with renal size (expressed as weight) reduced by approximately 25% and 44%, respectively, compared with contralateral uninjured kidneys (Figure 1C). PAS staining revealed increased inflammatory infiltrates and persistent tubular injury after IRI, characterized by the absence of brush borders, tubular cell flattening, and cast formation (Figure 1, D and E). The number of glomeruli per unit area was also increased as an indicator of kidney parenchymal loss and tubular atrophy (Figure 1F and Supplemental Figure 2) (38). In comparison, ischemic kidneys from entospletinib-treated mice were larger and displayed improved histopathologic findings, with reduced tubular injury and atrophy.

**Figure 2:**
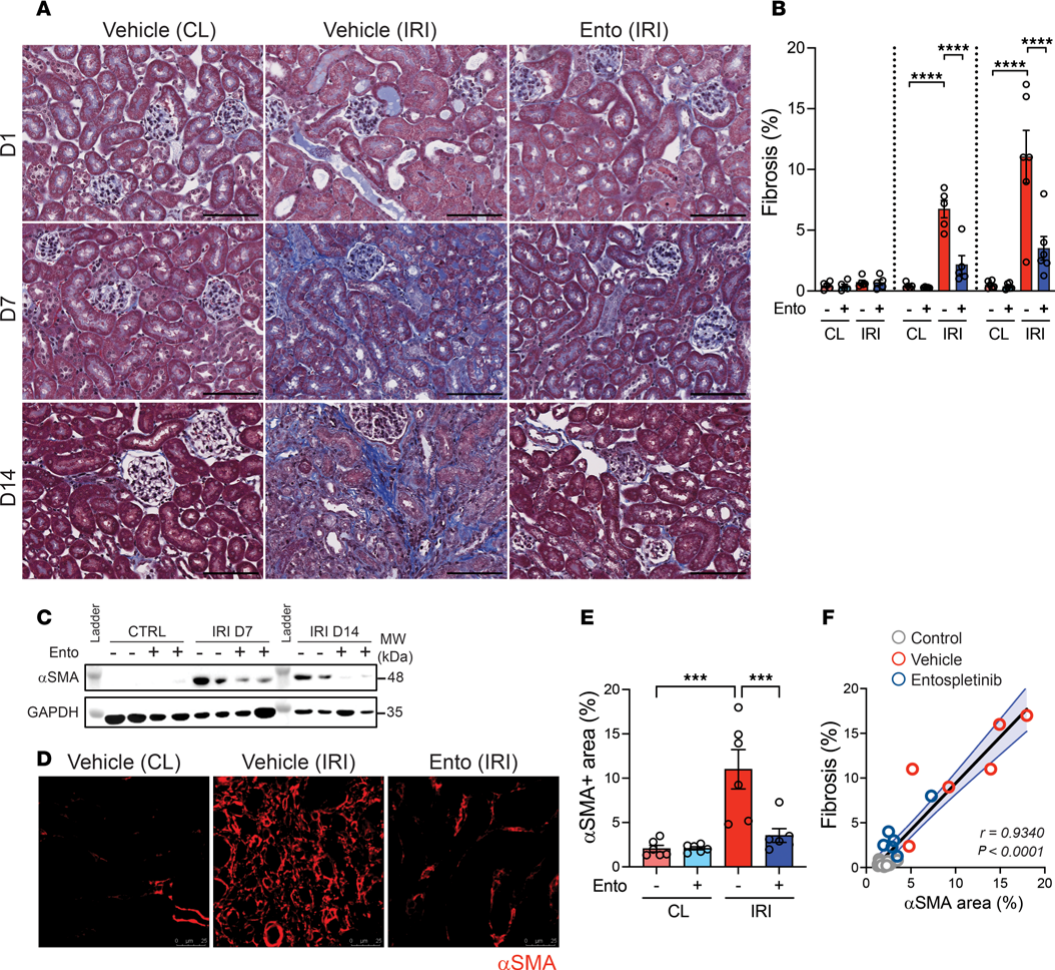
Entospletinib reduces fibrosis during AKI-to-CKD transition. (**A**) Representative Masson’s trichrome kidney histopathology from vehicle- and entospletinib-treated (Ento-treated) mice at 1, 7, and 14 days after ischemia reperfusion injury (IRI). Contralateral (CL) kidneys were used as controls. Scale bars: 100 μm. (**B**) Quantification of Masson’s trichrome–positive area/kidney (mean ± SEM, *n* = 5–6). (**C**) Immunoblotting of whole kidney lysates probing for α-smooth muscle actin (αSMA) and GAPDH at indicated treatments and time points. (**D**) Immunofluorescence microscopy probing for αSMA in kidneys from vehicle- or Ento-treated mice at 14 days after IRI. (**E**) Quantification of αSMA^+^ area (mean ± SEM. Each data point represents average of at least 3 fields of view/kidney, *n* = 5–6). (**F**) Correlation between αSMA^+^ area and percentage fibrosis (Masson’s trichrome–positive). Statistical analysis was performed using ANOVA followed by Bonferroni’s multiple-comparison test (**B** and **E**) and Pearson correlation test (**F**). ***P < 0.001; ****P < 0.0001.

Entospletinib also reduced renal fibrosis after IRI. As expected, kidneys on day 1 after IRI stained negative for fibrosis using Masson’s trichrome staining, despite tubular injury. By days 7 and 14, renal tubulointerstitial fibrosis increased in ischemic kidneys from vehicle-treated mice, indicating AKI-to-CKD transition (Figure 2A). Quantification revealed that entospletinib significantly reduced renal fibrosis area compared with vehicle-treated mice (6.74% ± 0.71% vehicle vs. 2.40% ± 0.55% entospletinib on day 7 and 13.00% ± 1.51% vehicle vs. 3.96% ± 1.05% entospletinib on day 14) (Figure 2B). Fibrosis was also assessed by immunoblotting and immunofluorescence microscopy. α-Smooth muscle actin (αSMA) protein expression and αSMA^+^ cells increased in the kidneys of vehicle-treated mice at 7 and 14 days after IRI in direct correlation with area of fibrosis, a response that was effectively inhibited by entospletinib (Figure 2, C–F).

**Figure 3:**
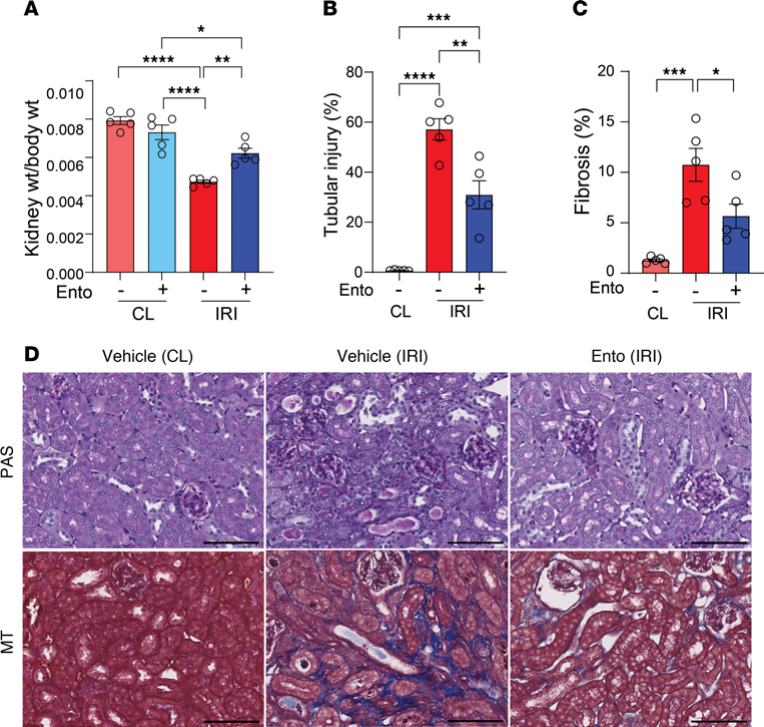
Entospletinib prevents AKI-to-CKD transition after established injury. Kidney pathology at 14 days following ischemia reperfusion injury (IRI) in mice treated with vehicle or entospletinib (Ento) starting 24 hours after surgery. Contralateral (CL) kidneys were used as controls. (**A**) Quantification of kidney weight/body weight ratio (mean ± SEM, *n* = 5). (**B**) Percentage of injured tubules/kidney (mean ± SEM, each data point represents average of at least 5 fields of view/kidney, *n* = 5). (**C**) Quantification of Masson’s trichrome–positive area/kidney (mean ± SEM, *n* = 5). (**D**) Representative periodic acid–Schiff (PAS) and Masson’s trichrome (MT) kidney histopathology for indicated treatments. Scale bars: 100 μm. Statistical analysis was performed using ANOVA followed by Bonferroni’s multiple-comparison test. *P < 0.05; **P < 0.01; ***P < 0.001; ****P < 0.0001.

To determine whether entospletinib was effective in established AKI (the more common clinical presentation in humans), the drug was initiated in mice at 24 hours after injury. At 14 days, mice treated with entospletinib displayed larger kidneys compared with vehicle-treated ischemic controls (kidney weight reduction ~40% control vs. 20% entospletinib) (Figure 3A). Consistent with the improved gross kidney changes, entospletinib-treated mice also showed reduced histopathologic tubular injury and interstitial fibrosis (Figure 3, B–D). Collectively, these data show that the SYK inhibitor entospletinib prevents IRI-induced AKI-to-CKD transition. Importantly, entospletinib is also effective when given after AKI, suggesting that it modulates pathways beyond the acute phase of kidney injury.

### Entospletinib promotes tubular integrity.

**Figure 4:**
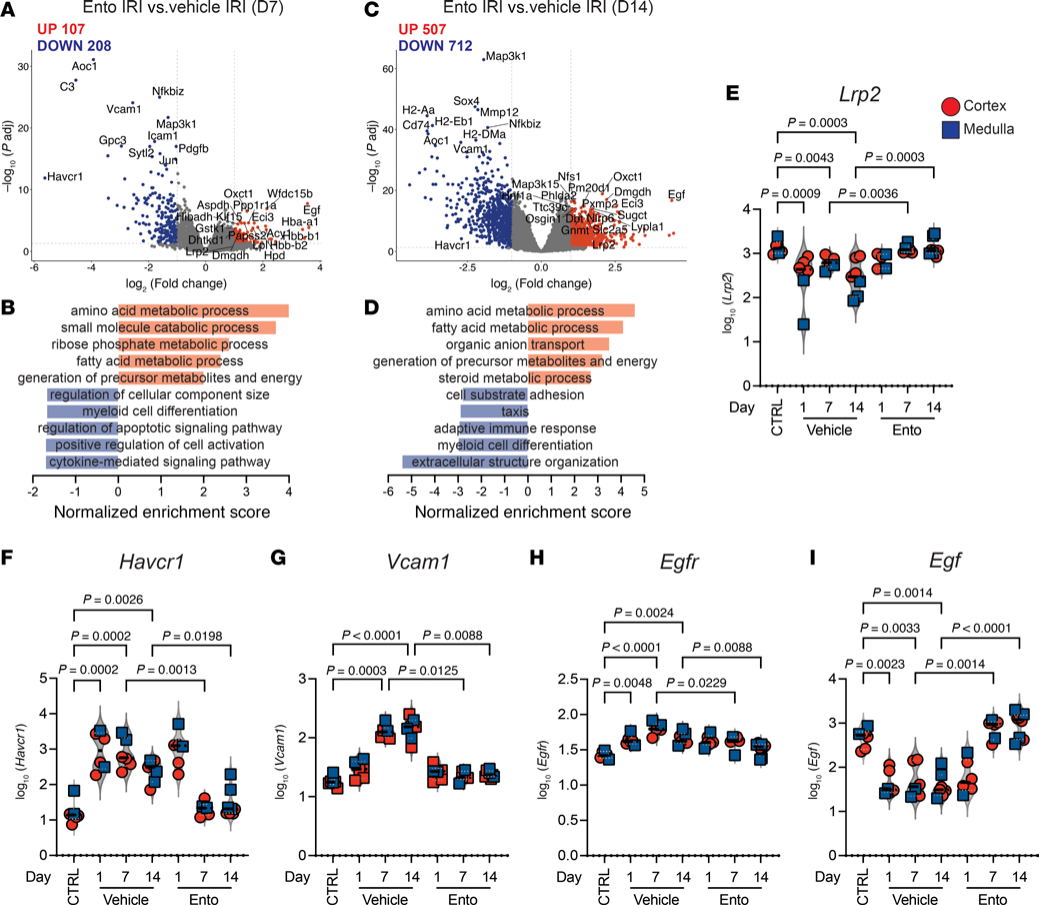
Entospletinib’s effects on tubular gene expression. Digital spatial profiling of CD10^+^ and PanCK^+^ tubules from the kidney cortex and medulla. (**A**–**D**) Volcano plots showing differentially expressed genes (adjusted *P* value < 0.05 and absolute log_2_[fold change] > 1) and gene set enrichment analysis using Gene Ontology (GO) when comparing tubules (PanCK^+^CD10^+^) from vehicle- and entospletinib-treated (Ento-treated) mice 7 and 14 days after ischemia-reperfusion injury (IRI). (**E**–**I**) The log_10_(normalized gene expression) for genes associated with tubular injury and repair during the AKI-to-CKD transition in vehicle- or Ento-treated mice: (**E**) *Lrp2*, (**F**) *Havcr1*, (**G**) *Vcam1*, (**H**) *Egfr*, and (**I**) *Egf*. Each red circle and blue square represents a region of interest in the cortex and medulla, respectively. Statistical analysis was performed using the Kruskal-Wallis test followed by Dunn’s multiple-comparison test.

To better characterize the phenotypic changes induced by entospletinib treatment in the tubular compartment during IRI, digital spatial profiling (DSP) using the GeoMx platform was employed. Kidney sections from vehicle- or entospletinib-treated mice on days 1, 7, and 14 after IRI were stained with anti-IBA1 (macrophages), anti-panCK, and anti-CD10 (tubular cells) to identify renal compartments and subsequent cell segregation (Supplemental Figure 3). The specificity of IBA1 and panCK/CD10 markers to differentiate between macrophage and tubular cell populations was confirmed by single-cell RNA-seq (scRNA-seq) (Supplemental Figure 4). Eighty-seven regions of interest (ROI) containing 12,661 cells were selected from both the cortex and medulla. Cells were then segregated according to their antibody label (areas of illumination, AOIs) followed by whole transcriptome sequencing of the 2 populations. Differential gene expression between IBA1- and panCK/CD10-defined AOIs confirmed good separation of macrophage and tubular cell compartments using this approach (Supplemental Figure 5 and Supplemental Data File 1). Differential gene expression analysis was next performed in panCK/CD10-defined segments to determine the effects of entospletinib on tubular phenotype following kidney injury. Few differentially expressed genes were detected in the tubular compartment of ischemic kidneys between vehicle- and entospletinib-treated mice on day 1 after IRI (Supplemental Figure 6). On days 7 and 14 after IRI, genes associated with “immune response,” “cell adhesion,” “cytokine-mediated signaling pathways,” “wound healing,” and “extracellular structure organization” were upregulated in ischemic kidneys of control mice, while genes related to “fatty acid metabolic process” and “generation of precursor metabolites and energy” were downregulated (Supplemental Figure 7). In contrast, entospletinib reversed the phenotype, upregulating genes linked to “fatty acid metabolic process” and “generation of precursor metabolites and energy” while downregulating genes associated with inflammation and “extracellular structure organization” (Figure 4, A–D). To further assess the effect of entospletinib on the tubular phenotype during AKI-to-CKD transition, the expression of genes associated with homeostasis and injury was compared (5). Over 14 days, IRI induced the upregulation of injury genes such as *Havcr1*, *Vcam1*, *Egfr*, *Lgals1*, *Lgals3*, and *Aoc1*, profibrotic genes *Ccn2* and *Vim*, inflammatory genes *Csf1*, *Ccl2*, *Il1b*, and *Cxcl2*, and downregulated epithelial homeostatic genes *Lrp2* and *Egf* (Figure 4, E–I, and Supplemental Figure 8). Although gene expression profiles were generally similar between the cortex and medulla, inflammatory and profibrotic genes tended to be higher in the medulla, which in this analysis included the outer medullary stripe. In contrast, treatment with entospletinib reduced the expression of all tubular injury–associated, profibrotic and proinflammatory genes while upregulating *Lrp2* and *Egf* to levels approaching uninjured control kidneys (Figure 4, E–I, and Supplemental Figure 8). Thus, in keeping with the improved pathologic findings, these results show that entospletinib prevents the molecular phenotype of AKI-to-CKD transition, while preserving a signature of intact tubules.

### Syk is expressed by leukocytes in the kidney.

**Figure 5:**
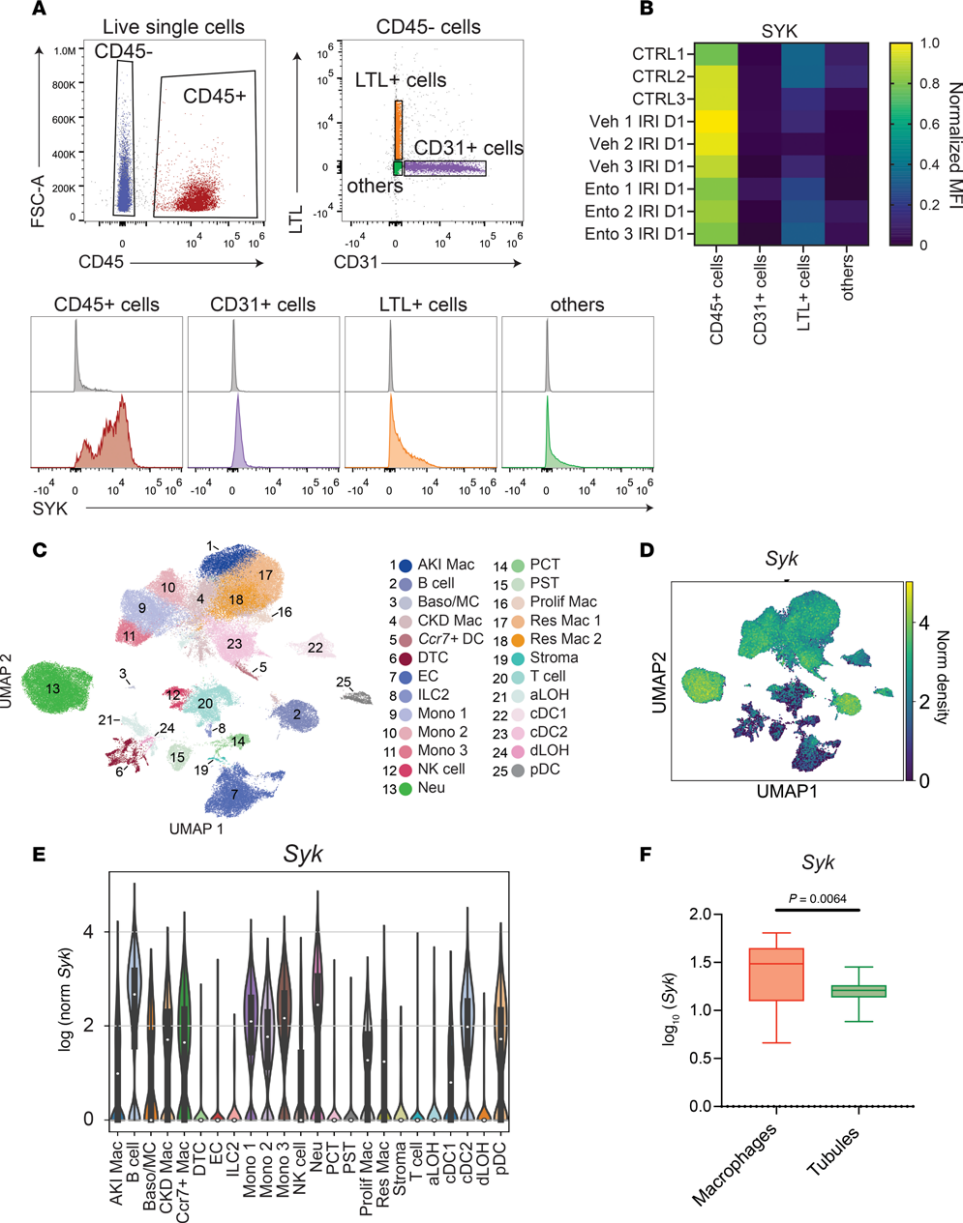
SYK expression in the kidney. (**A**) Flow cytometry scatter plots and histograms of cells isolated from normal kidney probing for SYK in CD45^+^ cells (leukocytes), LTL^+^ cells (tubular cells), CD31^+^ (endothelial cells), and “other” cells negative for these markers (gray represents the isotype control) (*n* = 2). (**B**) Heatmap of SYK expression analyzed by flow cytometry (MFI, mean fluorescence intensity) in different kidney cell populations isolated from 3 uninjured control (CTRL), vehicle- (veh), and entospletinib-treated (Ento-treated) mice at 24 hours following ischemia reperfusion injury (IRI). (**C**) Uniform manifold approximation and projection (UMAP) dimension reduction of 90,196 kidney cells with 4,000 genes. The UMAP contains 25 annotated clusters, including *Ccr7*^+^ dendritic cells (*Ccr7*^+^ DC), resident macrophages (res Mac 1 and res Mac 2), AKI- and CKD-associated macrophages (AKI and CKD Mac respectively), proliferation macrophages (Prolif Mac), monocytes (Mono 1, Mono 2, Mono 3), neutrophils (Neu), plasmacytoid DCs (pDC), conventional DCs (cDC1 and cDC2), B cells, T cells, natural killer cells (NK), innate lymphoid cells 2 (ILC2), basophils/mast cells (Baso/MC), endothelial cells (EC), ascending and descending loop of Henle cells (aLOH and dLOH respectively), distal convoluted tubular cells (DTC), proximal straight tubular cells (PST), proximal convoluted tubular cells (PCT), and stroma. (**D**) UMAP showing the expression pattern of *Syk* primarily in leukocyte populations. (**E**) Violin plot showing the expression of *Syk* in each annotated cluster. (**F**) *Syk* expression pattern in kidney cells by digital spatial profiling using log_10_(*Syk*) of macrophages versus tubules. Statistical analysis was performed using Student’s *t* test.

Previous studies have identified SYK as mainly leukocyte expressed in kidney IRI (30). Consistent with these reports, SYK was primarily detected in CD45^+^ leukocytes, with little to no expression in tubular cells (LTL^+^CD45^–^), endothelial cells (CD31^+^LTL^–^CD45^–^), or other cell types by flow cytometry on single-cell suspensions isolated from uninjured and injured mouse kidneys (Figure 5, A and B). scRNA-seq studies were next performed on kidney cells (enriched for leukocytes) isolated from mice undergoing IRI as described in the Methods (Supplemental Figure 9). Unbiased clustering identified 25 cell populations, which were manually annotated according to previously reported markers and CellMarker 2.0 and PanglaoDB (4, 5) databases (Figure 5C, Supplemental Figure 10, and Supplemental Figure 11A). In line with the flow cytometry results, scRNA-seq data confirmed high *Syk* expression in leukocytes, except for T lymphocytes, and less expression in tubular and other parenchymal cells (Figure 5, D and E). Comparison of *Syk* expression between macrophages and tubular cells in DSP data also showed higher expression in macrophages (Figure 5F). Finally, similar expression patterns were observed in external scRNA-seq datasets from mouse ischemic kidneys (4, 5) (data not shown). Therefore, SYK is primarily expressed by leukocytes during kidney injury, suggesting that entospletinib modulates the AKI-to-CKD transition via effects on this cell population.

### Entospletinib alters the immune cell composition of the injured kidney.

**Figure 6:**
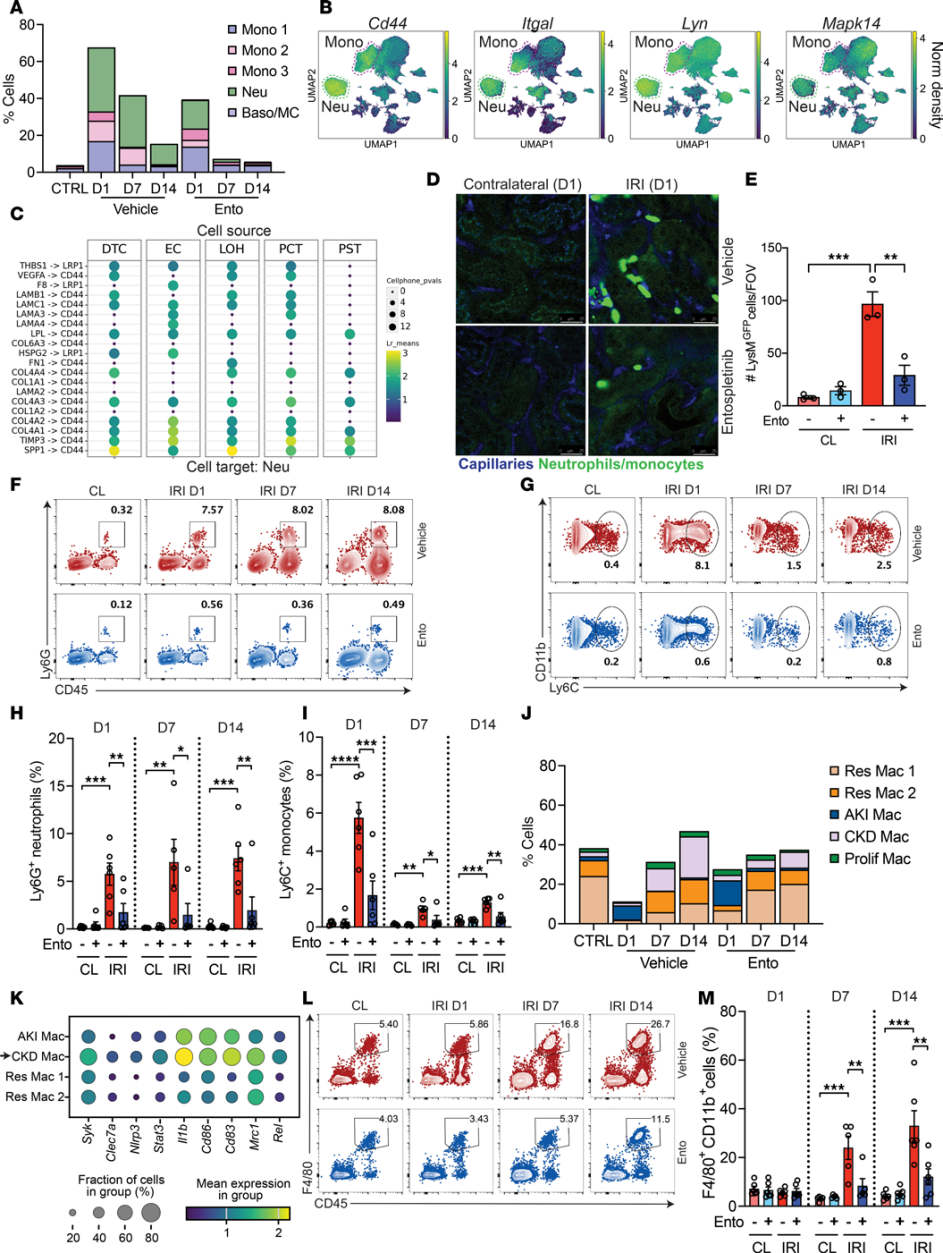
Entospletinib’s effects on kidney immune cell populations. (**A**) Proportions of infiltrating monocytes (Mono 1–3), neutrophils (Neu), and basophils/mast cells (Baso/MC) in kidneys of uninjured control (CTRL), vehicle-, and entospletinib-treated (Ento-treated) mice (scRNA-seq) over 14 days following ischemia-reperfusion injury (IRI). (**B**) UMAP showing the expression of *Cd44*, *Itgal*, *Lyn*, and *Mapk14* in kidney monocytes and neutrophils (outlined). (**C**) CellPhoneDB analysis of ligand-receptor interactions between distal tubular cells (DTC), endothelial cells (EC), loop of Henle cells (LOH), proximal convoluted tubular cells (PCT), proximal straight tubular cells (PST), and cell target neutrophils. (**D**) Intravital microscopy (IVM) in vehicle- and Ento-treated *LysM^gfp/gfp^* mice on day 1 after IRI. Uninjured contralateral (CL) kidneys were used as controls. Capillaries, blue; LysM-GFP^+^ leukocytes, bright green; tubules, dark green. Scale bars: 25 μm. (**E**) Quantification of kidney-infiltrating GFP^+^ leukocytes by IVM (mean ± SEM, each dot represents the average of 3 different fields of view/kidney, *n* = 3). Representative flow cytometry plots of renal neutrophils (CD45^+^CD11b^+^Ly6G^+^) (**F**) and monocytes (CD45^+^CD11b^+^Ly6C^hi^) (**G**) from vehicle- and Ento-treated mice over 14 days. Day 1 contralateral kidney is shown as a control. (**H** and **I**) Quantification of renal Ly6G^+^ neutrophils and Ly6C^+^ monocytes by flow cytometry (mean ± SEM, *n* = 5–6). (**J**) Renal macrophage subsets identified by scRNA-seq: resident (Res Mac), proliferating (Prolif Mac), AKI-associated (AKI Mac), and CKD-associated (CKD Mac) macrophages at baseline and over 14 days after IRI. (**K**) Dot plot of inflammatory genes in CKD and other macrophages. (**L**) Representative flow cytometry plots of CD45^+^F4/80^hi^ macrophages from vehicle- and Ento-treated mice over 14 days after IRI. Day 1 contralateral kidney is shown as a control. (**M**) Quantification of renal CD45^+^CD11b^+^F4/80^hi^ macrophages by flow cytometry (mean ± SEM, *n* = 5–6). Statistical analysis was performed using ANOVA followed by Bonferroni’s multiple-comparison test. *P < 0.05; **P < 0.01; ***P < 0.001; ****P < 0.0001.

Persistent or nonresolving inflammation is a critical driver of injury and maladaptive repair during AKI-to-CKD transition (4, 5). Since leukocytes are a likely target of entospletinib, scRNA-seq data were further analyzed to understand the effect of the drug on renal inflammation following IRI. Under normal conditions, the mouse kidney contains only a few monocytes and neutrophils (Supplemental Figure 12, A and B). On day 1 following IRI in vehicle-treated mice, the number of these leukocytes increased in the kidney. As AKI progressed to CKD, the number of neutrophils remained elevated at 14 days (Figure 6A and Supplemental Figure 12C). Neutrophils expressed high levels of *Cd44*, *Il1b*, *Cxcl2*, *Cxcr2*, *Mmp9*, and *Ptgs2* mRNA, suggesting that they contribute to nonresolving renal inflammation during AKI-to-CKD transition (Figure 6B, Supplemental Figure 11, and Supplemental Data File 2). On the contrary, the decrease in monocytes at 7 and 14 days likely reflected the phenotypic change to macrophages given the significant increase in macrophages and F4/80^+^ cells over that same time interval (Figure 6, A, J, L, and M, and Supplemental Figure 12). In entospletinib-treated mice, renal inflammation was subdued, with fewer monocytes and especially neutrophils infiltrating the injured kidneys over 14 days (Figure 6A and Supplemental Figure 12).

CD44 and ICAM-1 are key cell surface proteins involved in leukocyte recruitment to the kidney (25). SYK acts downstream of CD44 and Src family kinases in leukocytes to transition LFA-1 (*Itgal*) from a low- to high-affinity state, enabling firm adhesion to ICAM-1 on inflamed endothelium (39). *Cd44* and *Itgal* were predominantly expressed in monocytes and neutrophils, cells that were increased in number by IRI and decreased by entospletinib, whereas *Lyn*, *Mapk14*, and *Syk* were broadly expressed across all myeloid cells (Figure 5D and Figure 6B). Cell-to-cell communication inference using CellPhoneDB, with parenchymal cells as the source and monocytes/neutrophils as the target, revealed that *Cd44* on neutrophils and monocytes matched with several ECM genes expressed by tubular cells, such as *Spp1* and *Timp3*, and several collagen and laminin genes such as *Col1a1*, *Col6a3*, *Col4a1*, *Lama3*, and *Lamc1* (Figure 6C and Supplemental Figure 13). DSP analysis similarly revealed increased expression of *Spp1*, *Col1a1*, *Col3a1*, *Col4a1*, and *Icam1* as well as profibrotic factors such as *Ccn2* and *Tgfb1* in tubular cells following IRI (Supplemental Figure 8C and Supplemental Figure 14). Ischemic tubular cells also upregulated genes that participate in other aspects of leukocyte recruitment to the kidney such as *Il1b*, *Il34*, *Ccl2*, and *Cxcl2* (16, 40) (Supplemental Figure 8). These results are consistent with leukocyte homing to renal microenvironments containing ECM that are CD44 ligands (41). Compared with vehicle-treated mice, entospletinib decreased the expression of *Spp1*, *Col4a1*, *Col4a2*, *Icam1*, *Il1b*, *Il34*, *Ccl2*, and *Cxcl2*, suggesting that entospletinib impacts pathways that regulate the recruitment of neutrophils and monocytes to the injured kidney (Supplemental Figure 8 and Supplemental Figure 14).

To validate the effect of entospletinib on leukocyte recruitment, intravital microscopy and flow cytometry were employed. *LysM^gfp/gfp^* reporter mice, which express GFP predominantly in monocytes and neutrophils, underwent unilateral IRI after pretreatment with either vehicle or a single dose of entospletinib and ischemic kidneys assessed by multiphoton intravital microscopy at 24 hours when monocyte and neutrophil infiltration is the highest. Contralateral and uninjured kidneys showed very few adherent GFP^+^ leukocytes. Following IRI, significant recruitment of large GFP^+^ leukocytes was observed in the tubulointerstitial area of injured kidneys, with adherent and crawling cells present within peritubular capillaries and directly along tubules, consistent with the transcriptomic and computational analysis (Figure 6, D and E, and Supplemental Video 1). In contrast, entospletinib significantly reduced the number of infiltrating GFP^+^ cells in the kidney. In treated mice, the few GFP^+^ cells present in the ischemic kidneys were smaller and primarily circulating or briefly crawling within the peritubular capillaries, indicative of impaired cell activation and adhesion (Supplemental Video 2). The effect of entospletinib on neutrophil and monocyte recruitment, quantified by flow cytometry, aligned with the transcriptomic and intravital microscopy findings over 14 days after IRI. IRI induced a significant increase in kidney CD44^+^ neutrophils and monocytes at 24 hours as well as CD45^+^Ly6G^+^ neutrophils and CD45^+^CD11b^+^Ly6C^+^ monocytes over 14 days, effects that were significantly reversed by entospletinib (Figure 6, F–I, and Supplemental Figure 15). Therefore, the transcriptomic, intravital microscopy, and flow cytometry data collectively show that entospletinib inhibits early and late neutrophil/monocyte recruitment to the kidney following IRI.

The impact of entospletinib on dendritic cell and macrophage populations following IRI and AKI-to-CKD transition was next examined. scRNA-seq analysis demonstrated that for the most part, various dendritic cell populations increased over time after IRI, but entospletinib did not have a major impact on these cells (Supplemental Figure 16). On the other hand, entospletinib significantly altered the renal macrophage landscape. Cells annotated as resident macrophages, expressing genes such as *Cx3cr1*, *Arhgap22*, and *Tgfbr1*, were the largest leukocyte population in healthy kidneys (Figure 6J, Supplemental Figure 11, and Supplemental Data File 3). At 24 hours after IRI, a significant reduction in resident macrophages occurred in parallel with a transient increase in a distinct population annotated as AKI-associated macrophages. Besides the scavenger receptor *Cd36* (42), these AKI macrophages also expressed genes related to positive and negative immune modulation, and apoptotic cell clearance, including *Cxcl16*, *Creb5* (43), *Stab1* (44), *Dab2* (45), *Axl* (46), *Lilrb4* (47), and *C1qa* (48, 49) (Supplemental Figure 11B and Supplemental Data File 3). As AKI progressed to CKD, the resident macrophage population began to recover in vehicle-treated mice, while a population annotated as CKD-associated macrophages expanded and persisted over 14 days (Figure 6J). These nonresolving CKD-associated macrophages highly expressed genes such as *Mrc1*, *Clec7a*, *Nlrp3*, *Cd83*, *Cd86*, and *Syk* (Figure 6K, Supplemental Figure 11C, and Supplemental Data File 3). In contrast, entospletinib blunted the acute reduction in total kidney macrophages at 24 hours and preserved the resident population. At 7 and 14 days following IRI, resident macrophages increased further in entospletinib-treated mice compared with controls, while a notable reduction in the emergence of nonresolving CKD-associated macrophages was observed (Figure 6J). Flow cytometry corroborated the transcriptomic findings. IRI induced the progressive expansion of F4/80^hi^CD11b^+^ macrophages over 14 days of AKI-to-CKD transition, an effect that was attenuated by entospletinib (Figure 6, L and M). Importantly, the effects of entospletinib on neutrophil, monocyte, and macrophage populations in the injured kidney at 14 days were also observed by flow cytometry in mice that began treatment 24 hours after IRI (Supplemental Figure 17). Taken together, these results show that during IRI-induced AKI-to-CKD transition, entospletinib inhibits neutrophil/monocyte recruitment and nonresolving renal inflammation while preserving resident macrophage populations in the kidney.

### Entospletinib blocks proinflammatory macrophages and promotes a reparative phenotype.

**Figure 7:**
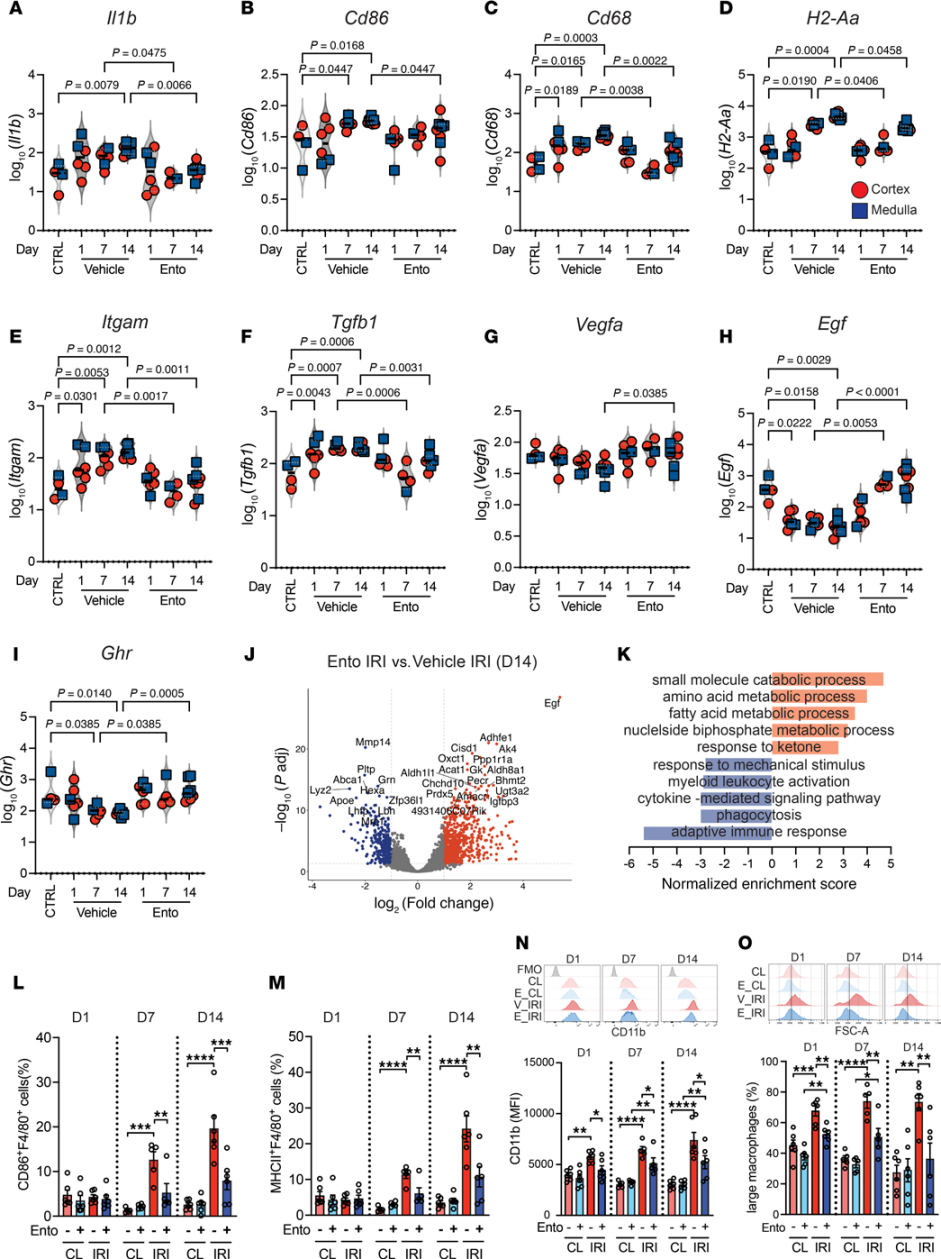
Entospletinib’s effects on macrophage gene expression. Digital spatial profiling of IBA1^+^ macrophages in the kidney cortex and medulla following ischemia-reperfusion injury (IRI) over 14 days. Log_10_(normalized expression) of (**A**) *Il1b*, (**B**) *Cd86*, (**C**) *Cd68*, (**D**) *H2-Aa*, (**E**) *Itgam*, (**F**) *Tgfb1*, (**G**) *Vegfa*, (**H**) *Egf*, and (**I**) *Ghr* in vehicle- or entospletinib-treated (Ento-treated) mice. Uninjured kidney was used as a control (CTRL). Each red circle and blue square represents a region of interest in the cortex and medulla, respectively. Statistical analysis was performed using the Kruskal-Wallis test followed by Dunn’s multiple-comparison test. (**J** and **K**) Volcano plots showing differentially expressed genes (adjusted *P* value < 0.05 and absolute log_2_[fold change] > 1) and gene set enrichment analysis using Gene Ontology (GO) comparing macrophages from vehicle- and Ento-treated mice 14 days after IRI. Flow cytometry of kidney leukocytes over 14 days after IRI demonstrating percentage of (**L**) CD86^+^F4/80^hi^ cells, (**M**) MHCII^+^F4/80^hi^ cells, (**N**) CD11b expression (MFI, mean fluorescence intensity), and (**O**) percentage of large macrophages. Contralateral (CL) kidneys were used as controls (mean ± SEM, *n* = 5–6). Statistical analysis was performed using ANOVA followed by Bonferroni’s multiple-comparison test. *P < 0.05; **P < 0.01; ***P < 0.001; ****P < 0.0001.

**Figure 8:**
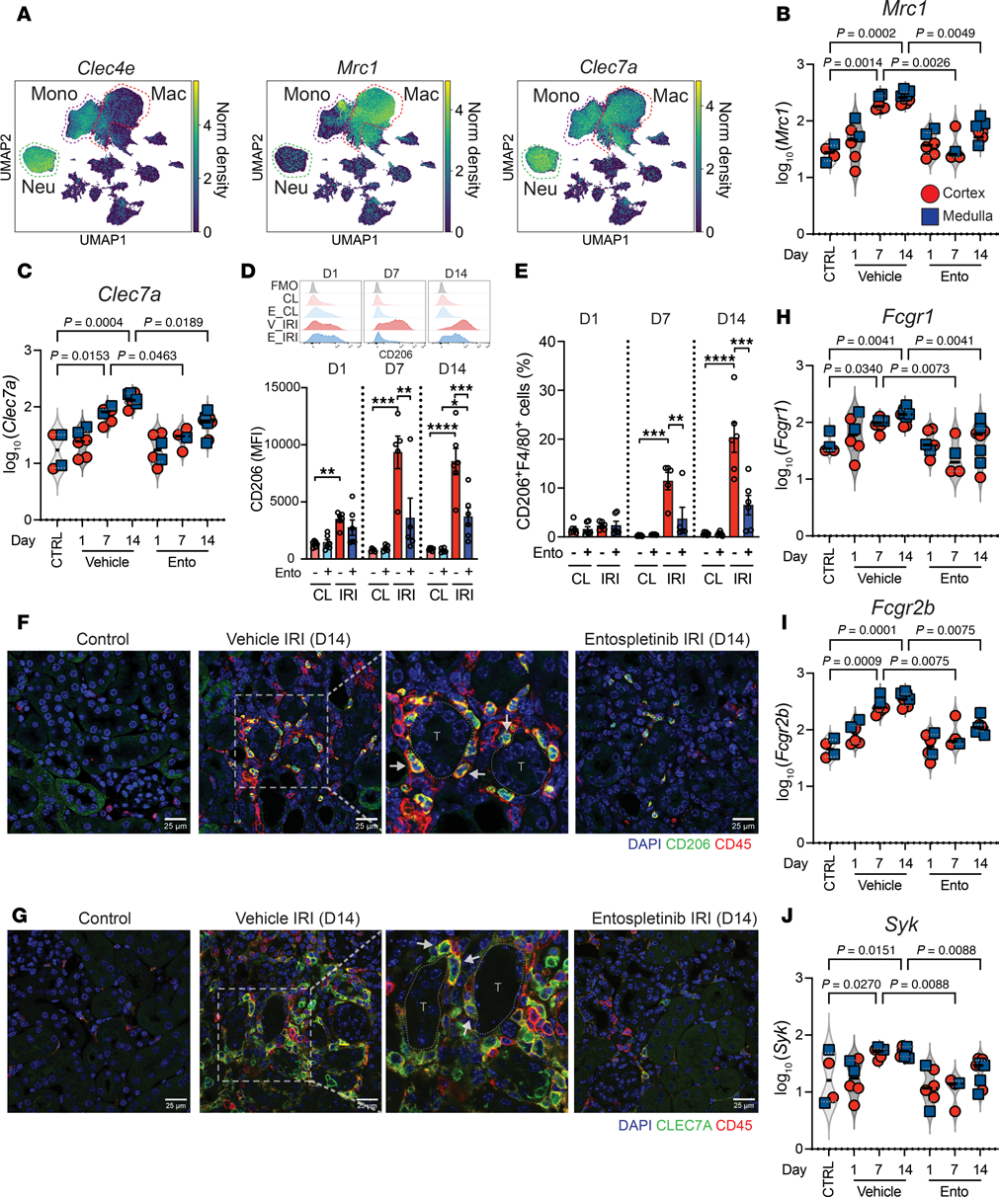
*Syk*-dependent pathways in AKI-to-CKD transition. (**A**) UMAP plot showing the distribution of *Clec4*, *Clec7a*, and *Mrc1* in kidney neutrophils, monocytes, and macrophages (scRNA-seq, outlined). The log_10_(normalized gene expression) of (**B**) *Mrc1* and (**C**) *Clec7a* in macrophages over 14 days after ischemia reperfusion injury (IRI) in vehicle- and entospletinib-treated (Ento-treated) mice (digital spatial profiling). Uninjured kidneys were used as controls (CTRL). Each red circle and blue square represents a region of interest in the cortex and medulla, respectively. Statistical analysis was performed using the Kruskal-Wallis test followed by Dunn’s multiple-comparison test. Flow cytometry showing (**D**) CD206 expression (mean fluorescence intensity, MFI) and (**E**) percentage of CD206^+^F4/80^hi^ cells in kidney leukocytes isolated from vehicle- and Ento-treated mice over 14 days following IRI. Contralateral (CL) kidneys were used as controls (mean ± SEM, *n* = 5–6). Statistical analysis was performed using ANOVA followed by Bonferroni’s multiple-comparison test. **P < 0.01; ***P < 0.001; ****P < 0.0001. Immunofluorescence microscopy probing for (**F**) CD206 and CD45 and (**G**) CLEC7A and CD45 in kidneys of vehicle- and Ento-treated mice 14 days following IRI. Arrows denote CD45^+^CD206^+^ and CD45^+^CLECL7A^+^ cells abutting tubules (T). Uninjured kidneys were used as controls. Scale bar: 25 μm. The log_10_(normalized gene expression) of (**H**) *Fcgr1*, (**I**) *Fcgr2b*, and (**J**) *Syk* in kidney macrophages over 14 days after IRI in vehicle- and Ento-treated mice (digital spatial profiling). Statistical analysis was performed using the Kruskal-Wallis test followed by Dunn’s multiple-comparison test.

SYK has been suggested to regulate macrophage phenotypes in vivo (23, 34) and thus the DSP transcriptomic data were probed to further understand the impact of entospletinib in this cell population. IRI activated macrophages and upregulated genes associated with a proinflammatory phenotype as early as day 1 after injury that persisted throughout the progression to CKD. Enrichment analysis of upregulated genes showed pathways associated with “cytokine-mediated signaling pathway,” “phagocytosis,” “myeloid cell activation,” “cell activation involved in immune response,” and downregulation of metabolic pathways involving “amino acids”, “fatty acids,” and “small molecules” (Supplemental Figure 18). These genes included *Il1b*, *Cd68*, *Itgam*, and *Cd86* but also genes typically associated with an antiinflammatory response such as *Mrc1* and *Tgfb1* (Figure 7, A–F, and Figure 8B). Entospletinib decreased macrophage inflammatory gene expression such as *Il1b*, *Il16*, *H2-Aa*, *Trem2*, *Ccl5*, *Ccl8*, *Ccl9*, *Tlr2*, *Tlr4*, *Tlr7*, *Cd68*, *Itgam*, *Cd86*, and *Igf1* while upregulating (or maintaining) genes associated with repair such as *Lilr4b*, *Vegfa*, *Vegfb*, *Igfbp3*, *Ghr*, and *Egf* (Figure 7, A–I and Supplemental Figure 19). Although some of the macrophage-expressed transcripts trended toward the renal cortex or medulla, clear regional changes in gene expression were not observed. Cross-validation against the scRNA-seq dataset confirmed the enrichment of these inflammatory and repair genes in macrophages (Supplemental Figure 11, C and D). Consistent with these changes in gene expression, a reciprocal switch in pathway enrichment was also seen (Figure 7, J and K, and Supplemental Figure 18, G and H). Flow cytometry was consistent with the proinflammatory macrophage mRNA signatures, demonstrating an increase in large macrophages expressing cell surface markers such as F4/80, CD86, MHCII, and CD11b over 14 days after IRI, changes that were attenuated by entospletinib (Figure 7, L–O and Supplemental Figure 20). Together, these data suggest that entospletinib significantly influences macrophage inflammatory and repair phenotypes induced by IRI and during the AKI-to-CKD transition.

SYK is activated downstream of DAMP-sensing C-type lectin receptors such as CLEC4E (Mincle, *Clec4e*) and CLEC7A (Dectin-1, *Clec7a*) that have been implicated in kidney disease (23, 27, 50). Similarly, macrophage mannose receptor-1 (CD206, *Mrc1*) can recognize collagen fragments and activate SYK via ITAM-bearing transmembrane FcRγ (51, 52). scRNA-seq revealed that *Clec4e* was primarily expressed by infiltrating monocytes/neutrophils (Figure 8A) that would allow sensing of DAMPs like SAP130 (Supplemental Figure 8I) and β-glucosylceramide, leading to SYK activation particularly during the acute phase of IRI (20, 23). Since neutrophil populations persisted over 14 days (Figure 6A), *Clec4e* signaling could therefore contribute to nonresolving inflammation observed in the AKI-to-CKD transition. Similarly, flow cytometry and immunofluorescence microscopy showed that *Clec7a* was expressed by macrophages, monocytes, and neutrophils, while *Mrc1* was exclusively expressed by macrophages, results that were verified in the DSP dataset (Figure 8, A–G and Supplemental Figure 20E). *Mrc1* and *Clec7a*, along with *Fcgr1*, *Fcgr2b*, and *Fcgr3* (genes needed for SYK activation) were upregulated in a time-dependent manner following IRI and during CKD progression. Again, macrophages in the medulla tended to express slightly higher levels of these genes but clear disparities between the renal cortex and medulla were not identified (Figure 8, B, C, H, and I, and Supplemental Figure 19). Additionally, *Syk* and other downstream molecules, such as *Grb2*, *Cdc42*, *Pak1*, *Nfkb1*, and *Nfkb2*, followed a similar expression pattern following IRI (Figure 8J and Supplemental Figure 19), consistent with coordinated inflammatory signal activation within macrophages during AKI-to-CKD transition.

**Figure 9:**
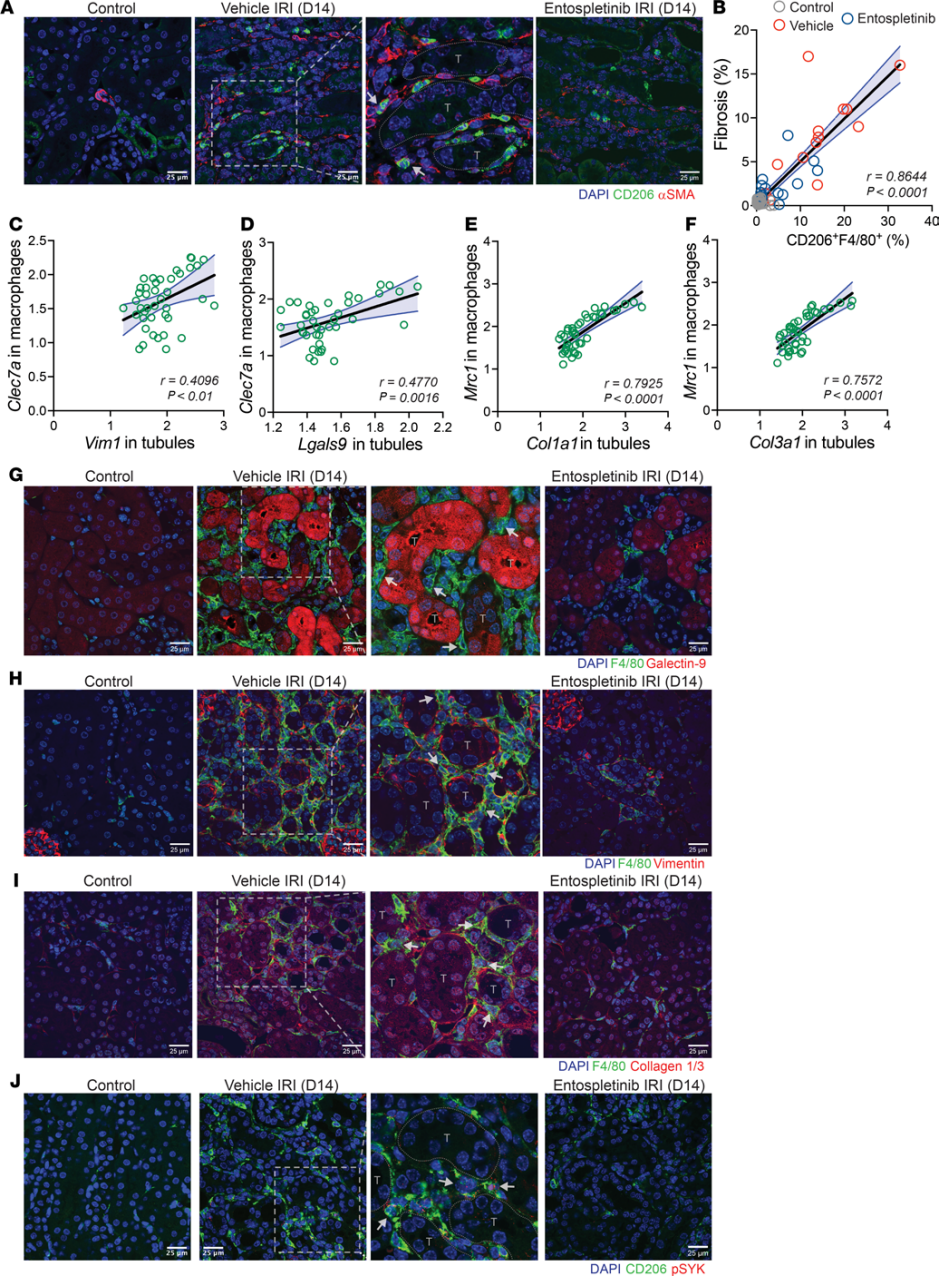
Macrophage interactions in the kidney during AKI-to-CKD transition. (**A**) Immunofluorescence microscopy in the kidneys of vehicle and entospletinib-treated mice at 14 days following ischemia reperfusion injury (IRI) probing for CD206 and αSMA. Uninjured kidney was used as a control. Arrows denote CD206^+^ macrophages interacting with αSMA^+^ cells in the interstitium. Scale bars: 25 μm. (**B**) The percentage of CD206^+^F4/80^hi^ macrophages correlated with the fibrosis area (%) calculated from whole kidney tissue sections in control, vehicle-, or entospletinib-treated mice following IRI (Pearson correlation test). The spatial correlation between *Clec7a* expressed in macrophages with (**C**) *Vim1* and (**D**) *Lgals9* expressed in tubular cells and *Mrc1* expressed in macrophages with (**E**) *Col1a1* and (**F**) *Col3a1* expressed in tubular cells in the same region of interest during IRI in vehicle-treated mice (digital spatial profiling, Pearson correlation test). (**G**–**I**) Immunofluorescence microscopy probing for F4/80, galectin-9, vimentin, and collagen 1/3 in the kidneys of vehicle- and entospletinib-treated mice at 14 days after IRI. Uninjured kidneys were used as controls. Arrows denote F4/80^+^ macrophages interacting with galectin-9–expressing tubules (T) and vimentin- and collagen 1/3–expressing tubules and interstitial cells. (**J**) Immunofluorescence microscopy probing for CD206 and phosphorylated SYK^Y519/520^ (p-SYK) in the kidneys of vehicle- and entospletinib-treated mice at 14 days after IRI. Uninjured kidneys were used as controls. Arrows denote p-SYK^+^CD206^+^ cells interacting with tubules and interstitial cells. Scale bars: 25 μm.

To further understand how SYK-dependent receptors such as CD206 and CLEC7A may be activated in the course of kidney injury, immunofluorescence microscopy and additional computational analysis were performed. Immunofluorescence microscopy demonstrated CD206^+^ and CLEC7A^+^ macrophages abutting injured tubules and CD206^+^ macrophages in close association with αSMA^+^ interstitial cells (Figure 8, F and G, Figure 9A, and Supplemental Figure 20E). Similarly, by measuring fibrotic area with Masson’s trichrome staining in tissue matched with our flow cytometry data, a significant direct correlation between CD206^+^F4/80^hi^ macrophages and fibrosis was observed (Figure 9B). These results suggested that DAMPs originating from parenchymal cells in the injured renal microenvironment may be driving macrophage activation and nonresolving inflammation. By using spatial information from the DSP datasets, the correlation between *Clec7a*- or *Mrc1*-expressing macrophages and their potential activating ligands during AKI-to-CKD transition was evaluated. A positive spatial correlation between *Clec7a*-expressing macrophages and injured tubular cells expressing *Vim* and *Lgals9* was identified (Figure 9, C and D), as well as a strong correlation between *Mrc1*-expressing macrophages and collagen-expressing tubular cells (Figure 9, E and F). These computational analyses were confirmed at the protein level using immunofluorescence microscopy that showed F4/80^+^ macrophages adjacent to galectin 9–expressing tubules, as well as vimentin- and collagen 1/3–expressing tubular and interstitial cells at 14 days following IRI (Figure 9, G–I). Importantly, many interstitial CD206^+^ macrophages, including those adjacent to injured tubules, expressed phosphorylated SYK (Figure 9J), reinforcing the premise that DAMPs released from injured cells in the renal microenvironment promote SYK and macrophage activation. In contrast, the relationship between macrophages, injured tubules, and fibrosis was greatly reduced or largely absent in the kidneys of mice treated with entospletinib. Entospletinib reduced the expression of *Mrc1*, *Clec7a* receptors, and associated FcRγ genes as well as the downstream signaling molecules, including *Grb2*, *Cdc42*, *Nfkb1*, *Nfkb2*, and *Syk* itself (likely due to a reduction in inflammatory signaling) throughout the AKI-to-CKD transition (Figure 8 and Supplemental Figure 19). Notably, *Cd4*-, *Foxp3*-, and *Ctla4*-expressing Treg populations that are also important for kidney repair were unaffected and remained elevated in entospletinib-treated mice (Supplemental Figure 21). Finally, entospletinib was also able to reverse macrophage activation over time in mice that started treatment 1 day after IRI (Supplemental Figure 22). Taken together, these data suggest that the SYK inhibitor entospletinib attenuates macrophage activation and disrupts a feedback loop of nonresolving inflammation and fibrosis while favoring a reparative microenvironment in the kidney.

## Discussion

In this study, we show that the SYK inhibitor entospletinib attenuates nonresolving inflammation and promotes renal repair in a model of IRI-induced AKI-to-CKD transition. The computational and experimental data support the premise that SYK is activated downstream of receptors such as CD206 in macrophages interacting with injured tubular and stromal cells in the kidney interstitium to drive nonresolving inflammation. Importantly, this study elucidates entospletinib’s mechanism of action in kidney injury and identifies it as a potential therapeutic agent to resolve renal inflammation and enhance repair for human AKI.

Immediately after AKI, immune cells are recruited to the kidney, guided by proinflammatory factors expressed from injured tubular cells and resident immune cells. Nonresolving inflammation is a key characteristic in the progression to CKD that contributes to the failure of kidney repair resulting in tubular atrophy and renal fibrosis. Our data suggest that SYK plays a major role propagating inflammation in the kidney downstream of CD206 and CLEC7A and are consistent with the previously reported role for the related C-type lectin receptor CLEC4E in AKI (23, 34). Maladaptive tubules during AKI-to-CKD transition are characterized by the high expression of *Vcam1*, *Havcr1*, and other proinflammatory genes combined with low *Lrp2* and *Egf* expression (5, 6, 17). These maladaptive tubules also express ECM proteins and DAMPs that are potential ligands for CD206, CLEC7A, and CLEC4E on infiltrating and resident macrophages (20, 23). The significant correlation between CD206- and CLEC7A-expressing macrophages with injured tubules as well as αSMA^+^ cells (that also produce ECM proteins and potential DAMPs) sheds additional light on how nonresolving renal inflammation is activated during kidney injury and the potential contribution of SYK in this immune mechanism. The attenuation of proinflammatory gene expression by entospletinib was associated with a reduction in the number of nonresolving CKD macrophages and a parallel reduction in αSMA^+^ cells, ECM, and growth factor gene expression. Our data therefore suggest that pharmacologic SYK inhibition with entospletinib appears to break the cycle of inflammation, injury, and fibrosis following IRI in the kidney.

SYK inhibition also enhanced repair pathways in the kidney. First, entospletinib blunted the maladaptive phenotype of injured tubules, decreasing *Vcam1* as well as the expression of numerous proinflammatory and profibrotic genes. Since tubular cells were found to express lower levels of *Syk*, the effect of entospletinib on this cellular compartment may have been indirect and related to the reduction in neutrophils and proinflammatory macrophages that expressed genes such as *Il1b*. A direct effect of entospletinib on tubules, however, cannot be ruled out. Second, entospletinib preserved resident macrophage and Treg populations that are needed for renal repair and maintained the expression of prorepair genes such as *Egf*, *Vegfa*, and *Ghr* (6, 12, 13, 15, 17). Thus, rather than directly activating repair pathways, entospletinib likely tipped the balance between inflammation/injury and repair, creating a more favorable environment for kidney recovery.

An effective therapeutic strategy for AKI and CKD should target inflammation and injury pathways as well as promote or activate renal repair. This study is one of the few to report a pharmacological agent that mechanistically targets nonresolving inflammation while enhancing repair pathways in the kidney. Entospletinib, a selective and potent second-generation SYK inhibitor, has completed several clinical trials for patients with hematological malignancies (53, 54). Entospletinib has a favorable short-term toxicity profile, and the human equivalent dose estimated from this study would be considerably lower than what is currently being used for lymphoma and leukemia (Supplemental Figure 1A) (37, 53). Furthermore, given the plasma drug concentrations achieved in this study that approach the EC_50_, entospletinib would be expected to have minimal off-target effects (36), although they cannot be completely excluded. Thus, a short course of entospletinib in patients at risk for AKI may be a feasible therapeutic approach that could be tested in a clinical trial.

Altogether, these results highlight the role of the immune system in the AKI-to-CKD transition and position SYK as a key modulator of nonresolving inflammation. SYK inhibition with entospletinib represents a potential therapeutic agent to improve outcomes for human AKI.

## Methods

### Sex as a biological variable.

Both male and female animals were used equally for general experiments. Male mice were used for scRNA-seq and DSP experiments due to the stronger IRI phenotype in this sex (55).

### Reagents.

Reagents used are listed in Supplemental Table 1.

### Animals.

WT and *LysM^gfp/gfp^* reporter C57BL/6 mice were raised from in-house breeding colonies. All the mice used in this study were obtained from colonies established and maintained at the University of Calgary.

### IRI model.

Renal IRI and AKI were induced by a surgical model of renal IRI in mice, as previously described (25). Briefly, mice were anesthetized using ketamine (200 mg/kg) and xylazine (10 mg/kg) supplemented with isoflurane. The left kidney was isolated through a small lateral incision, and a nontraumatic microaneurysm clip was applied to the renal pedicle under aseptic conditions. Ischemia was induced for 30 minutes at 37°C, followed by removal of the vascular clip and recovery of the animals with supplemental heat, hydration, and pain relief. Unmanipulated contralateral kidney or kidneys from healthy mice were used as controls. Mice were sacrificed on days 1, 7, and 14.

### In vivo treatment with entospletinib.

Mice were pretreated or not with an intraperitoneal injection of entospletinib (GS-9973, 20 mg/kg) or vehicle (DMSO) 30 minutes before IRI. No additional injections were used for IRI-induced AKI (animals euthanized on day 1). From day 1 until day 7, entospletinib (20 mg/kg) was injected daily and then every 2 days until euthanasia on day 14 as shown in Supplemental Figure 1.

### Entospletinib pharmacokinetics.

Three mice per time point were treated with a single intraperitoneal injection of entospletinib (GS-9973, 20 mg/kg) or left untreated (control, 0 hours). Blood was collected at 6, 12, and 24 hours after injection via cardiac puncture into citrate-coated tubes. Plasma was isolated by centrifugation at 10,000*g* for 10 minutes and stored at –80°C until analysis. Plasma entospletinib concentrations were measured at the Calgary Metabolomics Research Facility (CMRF) at the University of Calgary using a liquid chromatography–tandem mass spectrometry (LC-MS/MS) method as previously described (36, 56).

### Histopathology.

Samples for histology, including PAS and Masson’s trichrome staining, were processed by The Alberta Precision Laboratories, Calgary, Alberta, Canada. Images were generated with Aperio scanner (Leica Biosystems) by using a 40× objective. Tubular injury was assessed in the renal cortex and defined as tubular dilation, tubular atrophy, tubular cast formation, sloughing of tubular epithelial cells, and loss of the brush border. To quantitatively assess tubular injury, the total number of tubules and the number of damaged tubules in the renal cortex were measured from least 5 fields of view per kidney. The percentage of injury was calculated using the following formula: injury (%) = (damaged tubules/total tubules) × 100. QuPath (v0.5.0; https://qupath.github.io) was used to quantify the fibrotic area (Masson’s trichrome–positive area) in the whole kidney by using a smooth sigma of 1 and a threshold of 0.01 for the residual channel (57) (Supplemental Figure 2).

### Immunofluorescence microscopy.

Kidneys were harvested as indicated in each experiment and fixed in 10% (vol/vol) neutral-buffered formalin. Kidneys were dehydrated in a graded ethanol series and xylene, and finally embedded in paraffin. Following deparaffinization in xylene and rehydration in graded alcohol solution, sections (5 μm) were antigen retrieved with citrate buffer, pH 6 (Sigma-Aldrich). Sections were blocked and labeled with primary antibodies overnight. Tissues were washed and stained with secondary antibodies for 1 hour at room temperature, washed, and mounted with Prolong Gold antifade reagent with DAPI. All antibodies are described in Supplemental Table 1. At least 3 fields of view (FOV) per section were acquired with a Leica S8 confocal microscope and processed with native LAS X software (Leica Microsystems) or FIJI (ImageJ).

### Intravital microscopy.

Kidney intravital microscopy was performed as previously described (25). Briefly, mice were anesthetized and tail veins catheterized for drug and Qtracker 655 (5 μL) administration. The kidney was exteriorized using a lateral incision and extended over the heated imaging platform. Imaging was done with a Leica SP8 multiphoton confocal microscope and MaiTai Ti-Sapphire laser (Spectra Physics) at 800 to 850 nM excitation using predefined laser power and detector gain settings. Adherent leukocytes (LysM^–^GFP^+^) were manually counted in at least 3 fields of view.

### Immunoblotting.

Protein was isolated from mouse kidney tissue using radioimmunoprecipitation assay (RIPA) buffer (Sigma-Aldrich) supplemented with protease and phosphatase inhibitors. Protein samples were separated by SDS–polyacrylamide gel electrophoresis gels under reducing conditions. Proteins were transferred onto nitrocellulose membranes (GE Healthcare) and blocked for 1 hour with blocking solution before overnight incubation at 4°C with primary antibody (Supplemental Table 1). Blots were washed and incubated with appropriate secondary antibody conjugated to horseradish peroxidase at room temperature. Proteins were visualized with enhanced chemiluminescence Western blotting detection reagents (Bio-Rad) and digitally captured with a Chemidoc MP device (Bio-Rad).

### Preparation of single-cell suspensions.

Euthanized mice were perfused with chilled 1× PBS via the left ventricle. After removing the renal capsule and fat, kidneys were minced into approximately 1 mm^3^ cubes and digested using Multi-Tissue Dissociation kit (Miltenyi Biotec, 130-110-201). Up to 250 mg of tissue was digested with 50 μL of enzyme D, 25 μL of enzyme R, and 6.75 μL of enzyme A in 1 mL of RPMI and incubated for 30 minutes at 37°C. Digestion was deactivated by 10% fetal bovine serum (FBS). The suspension was passed through a 70 μm cell strainer. After centrifugation at 400*g* for 5 minutes, cell pellet was incubated with 1 mL of RBC lysis buffer on ice for 3 minutes and washed again. Cells were resuspended in PBS and passed through a 40 μm cell strainer. Cells were resuspended in staining buffer (1× PBS, 1% BSA, 2 mM EDTA) for further analysis.

### Flow cytometry.

Cells were resuspended in PBS and stained with Zombie-Aqua Fixable Viability Kit (BioLegend) for 10 minutes at room temperature. After a wash at 400*g* for 5 minutes, cells were resuspended in Fc block buffer (staining buffer + anti–mouse CD16/CD32) for 10 minutes at room tempertaure. Cells were washed and stained with the conjugated antibodies shown in Supplemental Table 1 using the gating strategy as shown in Supplemental Figure 23.

### scRNA-seq: cell processing and library preparation.

IRI was performed in 3 C57BL/6 mice/condition as described above, and kidneys were harvested and processed for single-cell suspension. Three mice with untouched kidneys were used as control. Single-cell suspensions from 3 mouse kidneys per condition were pooled and stained for 10 minutes with SYTOX Blue Dead Cell Stain (Invitrogen, S34857). Live leukocytes were enriched by fluorescence-activated cell sorting (FACS) by excluding SYTOX Blue^+^ cells and morphology (forward side scatter) (Supplemental Figure 9). Cell viability was further controlled by trypan blue exclusion. Cells with greater than 80% viability were fixed with an Evercode cell fixation kit (v2) (Parse Bioscience) according to the manufacturer’s protocols and were preserved at –80°C. Fixed cells were thawed and libraries were prepared by using Evercode WT (v2) (Parse Bioscience) according to the manufacturer’s protocols. Libraries were sequenced at 50K reads/cell by using the NovaSeq 6000 system with 5% PhiX at Novogene (https://www.novogene.com/us-en/).

### Raw data processing.

The resulting FASTQ files were analyzed using the Parse Biosciences data analysis pipeline (v1.1) (https://www.parsebiosciences.com), which demultiplexes reads and assigns cells and samples. Reads were aligned to the mouse genome (mm10).

### Data processing.

Data analysis was carried out using the SCANPY pipeline (58, 59). Quality control was performed to exclude low-quality and dead cells. Only genes expressed in at least 5 cells and cells that contained (a) more than 300 genes, (b) less than 20,000 counts, (c) less than 25% mitochondrial genes, and (d) less than 1.5% ribosomal genes were included in our analysis. The data were subsequently normalized and transformed logarithmically using the SCANPY package (v1.10.1). This process was performed with the functions scanpy.pp.normalize_per_cell (setting counts_per_cell_after = 10e4) and scanpy.pp.log1p. The scVI algorithm was applied for batch effect correction and dimensionality reduction (deep generative modeling for single-cell transcriptomics) (60).

Highly variable genes were calculated with sc.pp.highly_variable_genes function (min_mean = 0.0125, max_mean = 3, min_disp = 0.25). Then, the Leiden community detection was embedded into a k-nearest-neighbor graph. Afterwards, we created uniform manifold approximation (UMAP) embeddings to visualize this most relative neighbor graph using a resolution of 1.

In order to annotate the cell identity, we performed a differential gene expression analysis by using the sc.tl.rank_genes_group function in SCANPY with Wilcoxon’s method and obtained a list of the most representative genes from each population. We used these genes as input in CellMarker 2.0 (http://117.50.127.228/CellMarker/), which can infer the cell ID. Cell clusters were manually annotated based on CellMarker 2.0 prediction and by using previously reported markers for each cell type and PanglaoDB (https://panglaodb.se/) and WEB-based Gene Set Analysis Toolkit (https://www.webgestalt.org/option.php).

### Cell-to-cell communication.

Several computational tools were employed to infer cell-cell communication from single-cell transcriptomic data by applying the CellPhoneDB v2 method (https://www.sc-bestpractices.org/mechanisms/cell_cell_communication.html) (61).

### DSP: sample and library preparation and RNA-seq.

Samples were processed by the Applied Spatial Omics Centre (ASOC) at the University of Calgary. Digital spatial RNA profiling for FFPE samples from 2 control (CTRL), 2 vehicle IRI day 1 (V IRI D1), 2 entospletinib IRI day 1 (E IRI D1), 2 vehicle IRI day 7 (V IRI D7), 2 entospletinib IRI day 7 (E IRI D7), 2 vehicle IRI day 14 (V IRI D14), and 2 entospletinib IRI day 14 (E IRI D14) kidney tissue sections were conducted using the GeoMx Digital Spatial Profiler (NanoString) (62). Slides were prepared by baking in a drying oven for 3 hours at 60°C and then processed following the Nanostring GeoMx Digital Spatial Profiler slide preparation (MAN-10150) and NGS readout (MAN-10153) manual. Briefly, slides were deparaffinized with xylene and then rehydrated through a graded ethanol series. Targets were exposed by incubating with 1× Tris-EDTA buffer, pH 9 followed by 1 μg/mL proteinase K digestion. Next, Mouse Whole Transcriptome Atlas (WTA) Probes (NanoString Technologies) were hybridized to the tissues at 37°C for 16 to 24 hours. Excess and off-target probes are removed by 2× SSC/50% formamide. After washing, the slides were blocked in Buffer W and stained with morphological markers for 1 hour at room temperature. The fluorescently conjugated morphology markers used are as follows: SYTO 13 nuclei acid stain at 1:50 dilution (GMX-MORPH-NUC-12, NanoString), PanCK-AF532 at 1:50 dilution (AE-1/AE-3, NBP2-33200AF532, Novus), IBA1-AF594 at 1:100 dilution (E4O4W,48934, Cell Signaling Technology), and CD10-AF647 at 1:50 dilution (EPR22867-118, ab261729, Abcam). Excess markers were removed with 2 washes in 2× SSC. Stained slides were loaded onto a GeoMX Digital Spatial Profiler and scanned. During the study, at least 1 ROI in the medulla and 2 ROIs in the cortex were selected using morphological markers as guides and glomeruli avoided. For further analysis, 2 segmented AOIs were chosen: macrophages were identified as IBA1^+^ cells, and tubular cells were identified as either CD10^+^pan-CK^+^ double-positive cells or only Pan-CK^+^ cells. RNA-ID and UMI-containing oligonucleotide tags were UV-cleaved from the WTA probes within the chosen ROIs collected in a 96-well collection plate. For library preparation, Illumina i5 and i7 dual-indexing primers were added to the oligonucleotide tags during PCR to uniquely index each AOI. AMPure XP beads (Beckman Coulter) were used for PCR purification. Library concentration was measured using a Qubit fluorometer (Thermo Fisher Scientific), and quality was assessed using a Bioanalyzer (Agilent Technologies). Sequencing was performed on an Illumina NextSeq 2000 (Illumina Inc.) and fastq files were processed into gene count data for each sample using the GeoMx NGS Pipeline.

### Data processing and analysis.

Data preprocessing was performed by the ASOC at the University of Calgary based on the GeoMx Workflows vignettes (https://www.bioconductor.org/packages/release/workflows/vignettes/GeoMxWorkflows/inst/doc/GeomxTools_RNA-NGS_Analysis.html). R software (v4.3.1; https://www.r-project.org) was used to preprocess the GeoMx Digital Spatial Profiler data and the following packages were used: NanoStringNCTools (v1.8.0), GeoMxWorkflows (v1.6.0), GeomxTools (v3.4.0), stringr (v1.5.1), yaml (v2.3.7), dplyr (v1.1.3), scales (v1.2.1), ggplot2 (v3.4.3), ggpubr (v0.6.0), ggsankey (v0.0.99999), plotly (v4.10.3), reshape2 (v1.4.4), and DescTools (v0.99.50) (https://nanostring.com/products/geomx-digital-spatial-profiler/geoscript-hub/). Sequencing quality and adequate tissue sampling for every 94 ROI/AOI segment were assessed with the NanoString recommended parameters. Background signal was evaluated by calculating the limit of quantification (LOQ) using the negative probes in the WTA panel that did not target mRNA and low-performing probes were removed. Next, gene-level aggregation was calculated for multiple probes that target a single gene. Then, the LOQ was calculated and segments with exceptionally low signal were removed. Finally, the gene detection rate was calculated and genes below the threshold of 0.05 were filtered out. Upper quartile normalization was applied to the raw count matrix and 2 samples were excluded as outliers in principal component analysis and expression distributions before normalization. The thresholds used in this analysis are listed in Supplemental Table 2.

### Differential gene expression analysis.

Differential gene expression was performed with R package DESeq2 by following the instruction of Love et al. (63). Each ROI from macrophage and/or tubular segments was considered as a replicate in the analysis. Tables with the results can be found in Supplemental Data File 2.

### Enrichment analysis.

DESeq2 output were used to generate.rnk files and used in WEB-based Gene Set Analysis Toolkit (https://www.webgestalt.org/option.php). Additionally, up- and downregulated genes that had an adjusted *P* value of less than 0.05 and absolute log_2_(fold change) greater than 1 were used as input in the online tool ShinyGO 0.8 (http://bioinformatics.sdstate.edu/go/) (64). Gene Ontology (GO) Biological Process was used for the analysis, together with a cutoff of false discovery rate (FDR) less than 0.05. Pathway size: min = 2 and max = 5000. The top 10 pathways sorted by –log(FDR) are represented in the figures of this work.

### Statistics.

Data are shown as mean ± SEM. GraphPad Prism (v10.0.0) was used to perform all statistical analyses. Results were analyzed for statistical variance using unpaired 2-tailed Student’s *t* test, 2-tailed Mann Whitney test, Friedman’s, Kruskal-Wallis, or 1-way analysis of variance (ANOVA) with Dunn’s or Bonferroni’s post hoc test as appropriate. Results at *P* less than 0.05 were considered statistically significant.

### Study approval.

All procedures and protocols regarding animal experiments were reviewed and approved by the University of Calgary Animal Care Committee (Animal protocol: AC22-0201).

### Data availability.

Data for all experiments are available in the Supporting Data Values file. The complete scRNA-seq transcriptomic and DSP datasets are available in the NCBI Gene Expression Omnibus (https://www.ncbi.nlm.nih.gov/geo; GEO GSE299409 and GSE299516).

## Author contributions

EEE designed and conducted the majority of experiments, analyzed data, and wrote the manuscript. AL, SGB, and GA conducted experiments, acquired data, and reviewed the manuscript. A Derakhshani, A Dufour, and NAB contributed to the computational analysis. CNJ provided support and infrastructure for the intravital microscopy experiments. JC cosupervised EEE and SGB, designed experiments, and reviewed the manuscript. DAM oversaw the design and conduct of the entire project and wrote the manuscript.

## Supplementary Material

Supplemental data

Supplemental data set 1

Supplemental data set 2

Supplemental data set 3

Unedited blot and gel images

Supplemental video 1

Supplemental video 2

Supporting data values

## Version 1 08/22/2025

Electronic publication
