# Supplementary material for: The spleen tyrosine kinase inhibitor entospletinib resolves inflammation to promote repair following acute kidney injury
Source: JCI Insight. 2025 Aug 22;10(16):e189601. doi: 10.1172/jci.insight.189601 (PMC12406726; doi:10.1172/jci.insight.189601)
Supplement: Supplemental data [file jciinsight-10-189601-s013.pdf]

Supplemental Figures

The Spleen Tyrosine Kinase Inhibitor Entospletinib Resolves Inflammation to Promote Repair Following Acute Kidney Injury

Esteban E. Elias[1], Arthur Lau[1], Sisay Belay[1], Afshin Derakhshani[2], Graciela Andonegui[1], Craig Jenne[,2], Antoine Dufour[2], Nathan Bracey[1], Justin Chun[1] and Daniel A. Muruve[1,3]

[1]Department of Medicine, [2]Department of Microbiology, Immunology and Infectious Diseases, Snyder Institute for Chronic Diseases, University of Calgary, Alberta, Canada.

Supplemental Figure 1

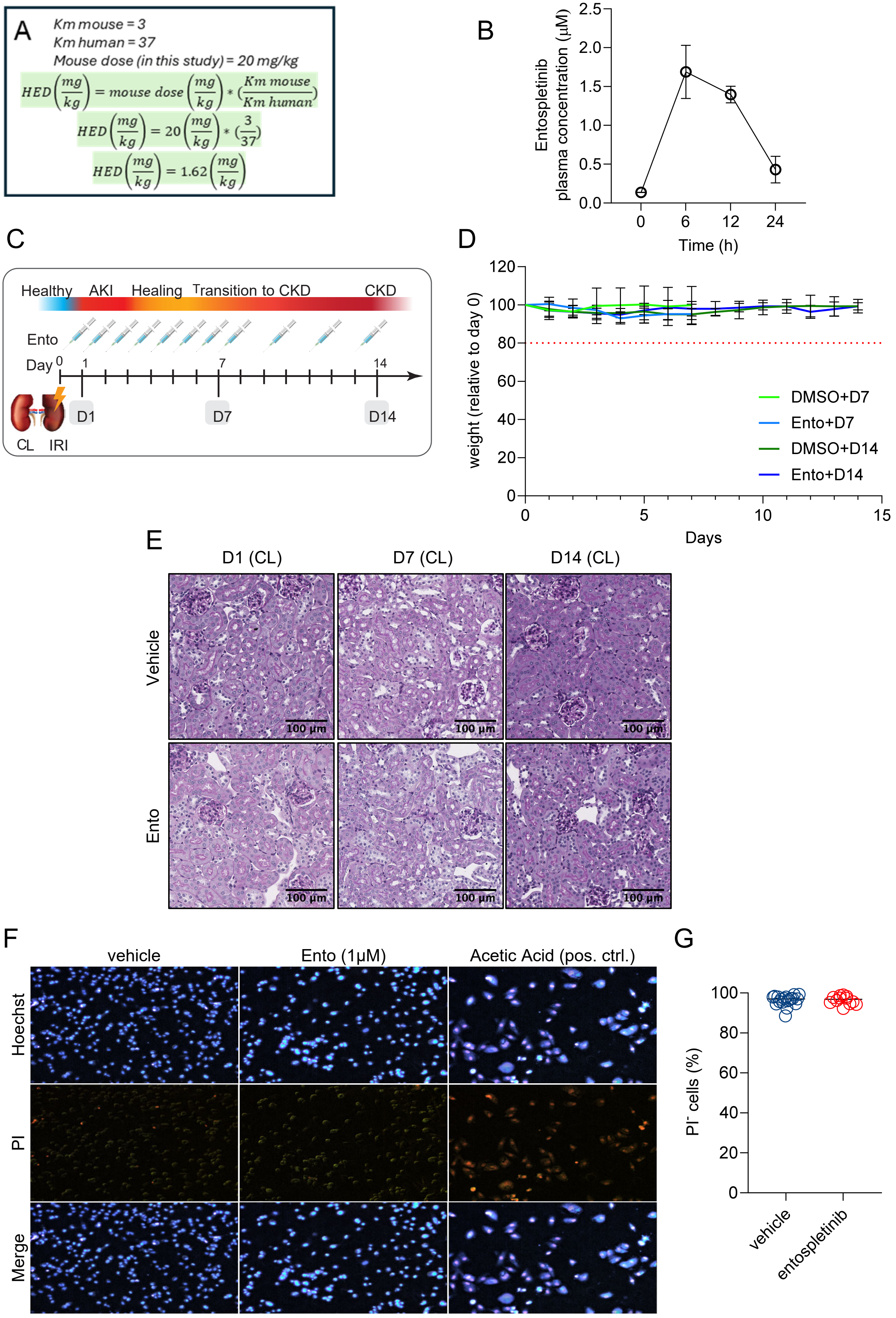

Supplemental Figure 1. In vivo and in vitro cytotoxic effect of Entospletinib. (A) Calculation of human equivalent entospletinib dose from dose used in this study. **(B)** 24 hour pharmacokinetics of entospletinib 20 mg/kg administered intraperitoneally in mice. **(C)** C57BL/6 WT mice were treated with entospletinib 30 minutes before ischemia reperfusion injury (IRI) as described in the methods. **(D)** Body weight was monitored daily before and after IRI surgery, normalized to day 0 (pre-surgery). No significant differences were observed between groups (ANOVA). **(E)** PAS staining comparing contralateral kidneys from vehicle and entospletinib-treated mice (n=5-6). Human TECs (hTECs) were cultured to ~90% confluence and treated with entospletinib for 24 hours. Cell viability was assessed using Hoechst-33342 and propidium iodide (PI) staining under an epifluorescence microscope (10x magnification). hTECs treated with acetic acid for 10 min served as a positive control. At least 4 regions of interest (ROIs) were analyzed per condition. **(F)** Representative PI staining image for each condition. **(G)** Mean ± SEM of the percentage of viable cells per ROI. Each dot represents an independent ROI (n=3). Statistical analysis using a two-tailed t-test showed no significant differences between groups.

Supplemental Figure 2

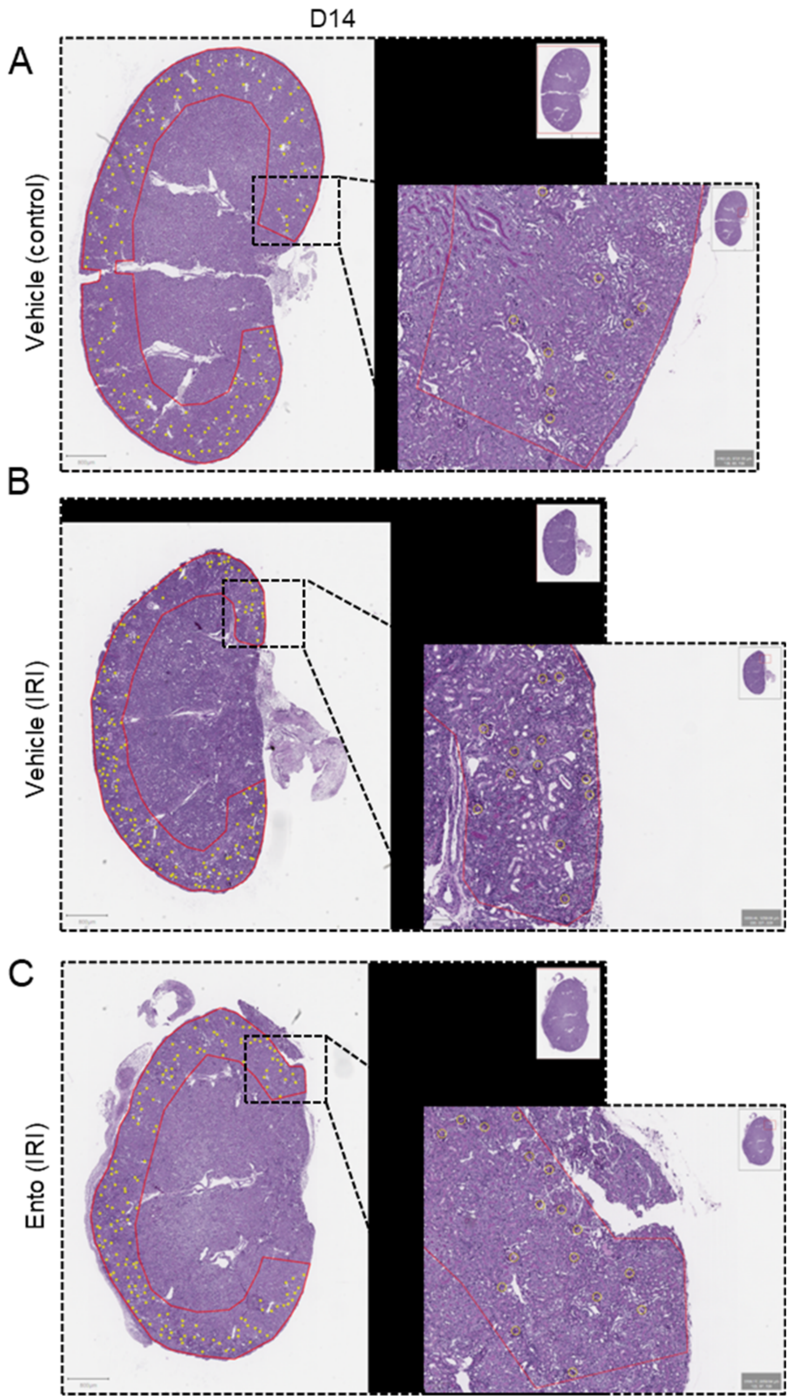

Supplemental Figure 2. AKI-to-CKD model and glomerular density quantification. C57BL/6 mice were intraperitoneally injected with entospletinib (ento) or vehicle (DMSO) 30 min before and after surgery at the indicated timepoints. Ischemia-reperfusion injury (IRI) was induced in the left kidney, while the right kidney served as a control. Kidneys were collected on days 1, 7, and 14 (D1, D7, D14). PAS-stained kidney images were acquired at 40x magnification and analyzed with QuPath. Cortical areas were delineated, glomeruli manually counted, and glomerular density calculated. Representative images show **(A)** control, **(B)** ischemic, and **(C)** entospletinib-treated kidneys at D14.

Supplemental Figure 3

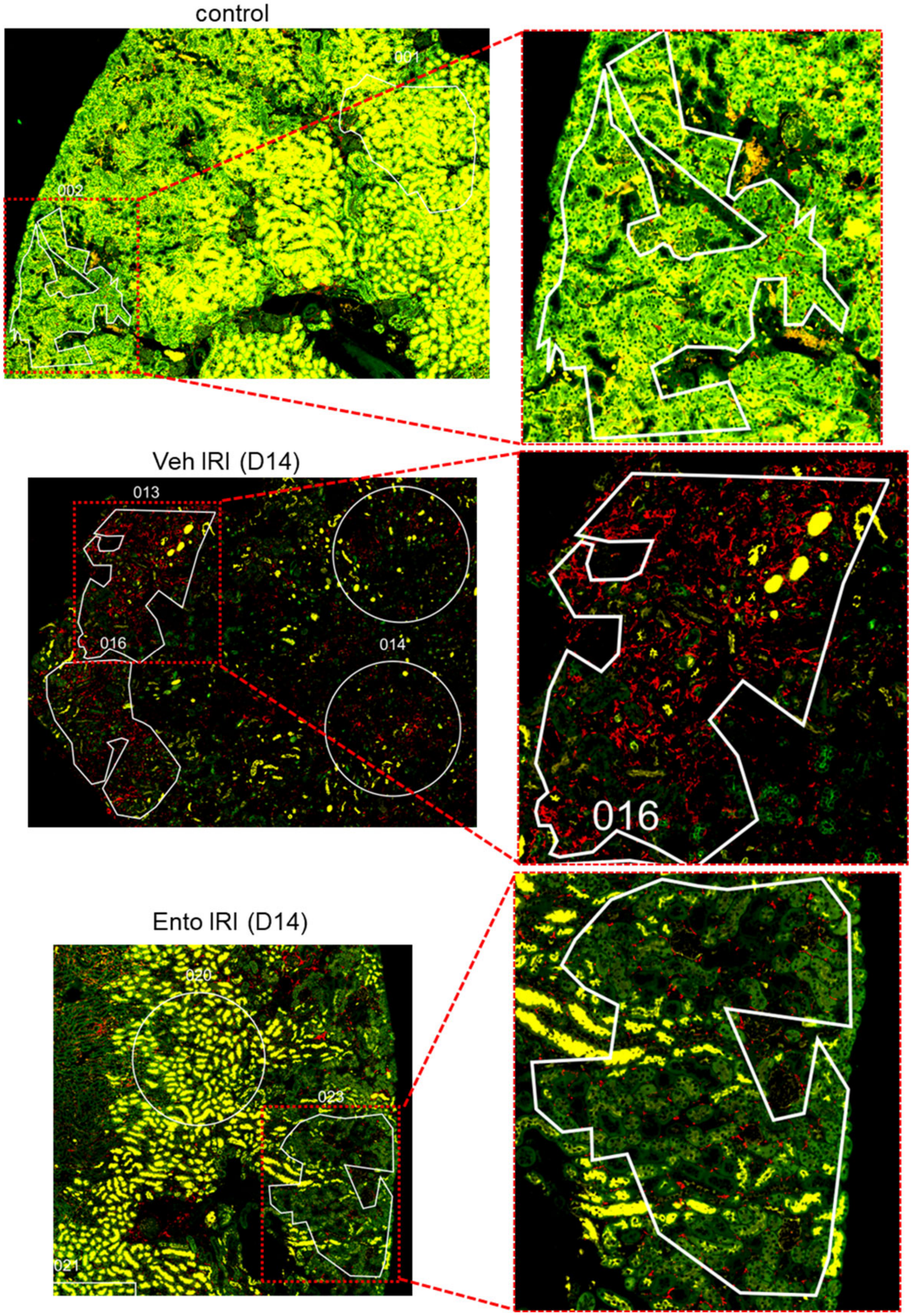

Supplemental Figure 3. Example of a DSP region of interest (ROI). Two FFPE kidneys per condition were stained with anti-CD10 (yellow) and anti-panCK (green) for tubules, and anti-IBA1 (red) for macrophages in the cortex and medulla. Within the region of interest (ROI), cells are then segregated into areas of illumination (AOI) defined by antibody labeling, followed by whole transcriptome sequencing of the segregated cell populations.

Supplemental Figure 4

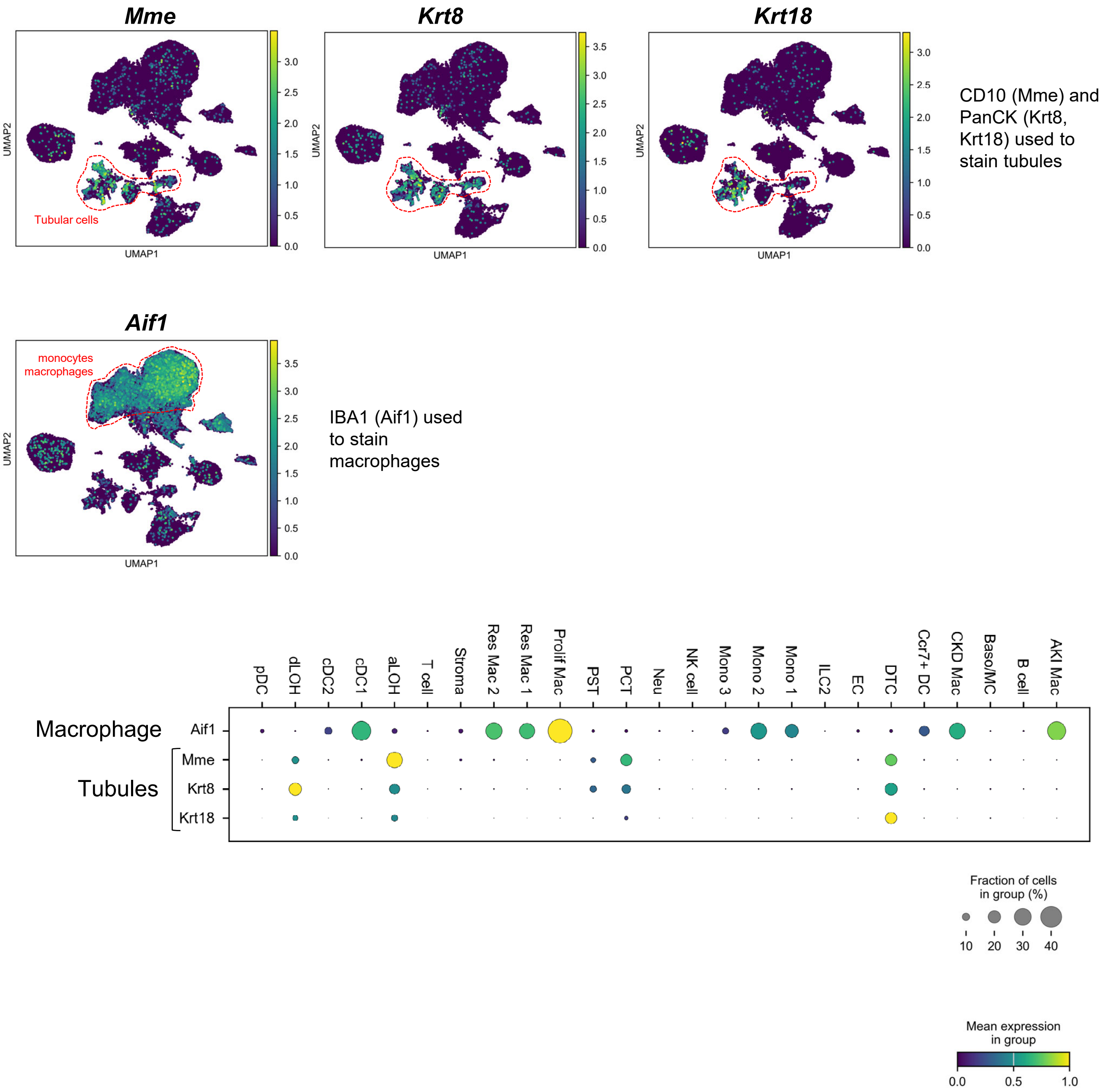

Supplemental Figure 4. Cell type–specific expression of DSP marker genes in scRNA-Seq data. Genes used for digital spatial profiling (DSP) were validated in scRNA-seq to confirm specificity: *Aif1* for macrophages; *Mme*, *Krt8*, and *Krt18* for tubular epithelial cells. **(A)** UMAP plots showing the expression of *Mme* (CD10), *Krt8*, *Krt18* (cytokeratins), and *Aif1* (IBA1). **(B)** Dot plot depicting the expression of these markers across all identified cell types.

Supplemental Figure 5

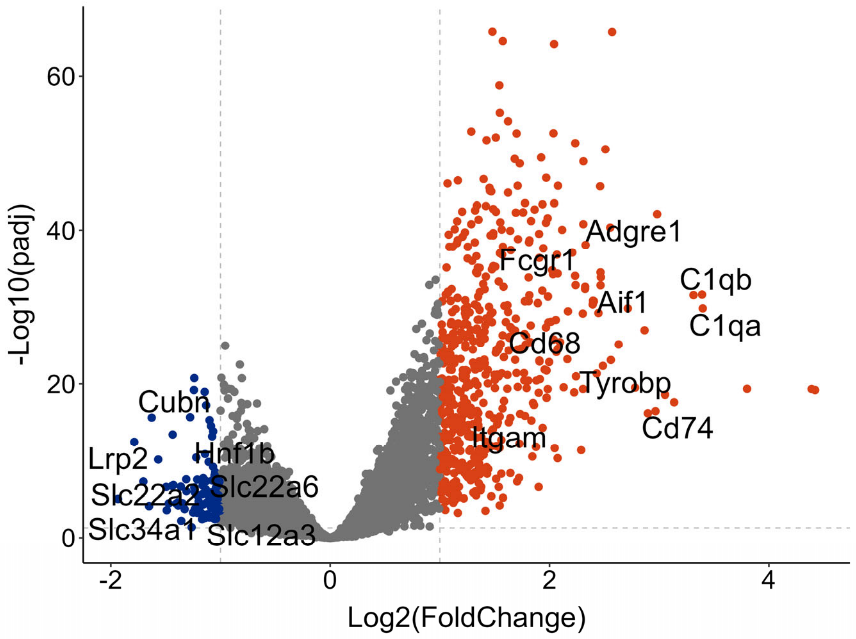

Supplemental Figure 5. Differential gene expression between DSP segments. Digital spatial profiling (DSP) used IBA1 to stain macrophages and CD10/PanCK to stain tubules in kidney tissue sections. Regions of Interest (ROIs) in the cortex and the medulla were chosen (2 sections/condition) and cell populations segregated based on their fluorescent label followed by whole transcriptome RNA sequencing. Volcano plots showing differentially expressed genes (padj < 0.05 and abs (log2 Fold Change) > 1) when comparing tubular cells (PanCK+CD10+) vs renal macrophages (IBA1+).

Supplemental Figure 6

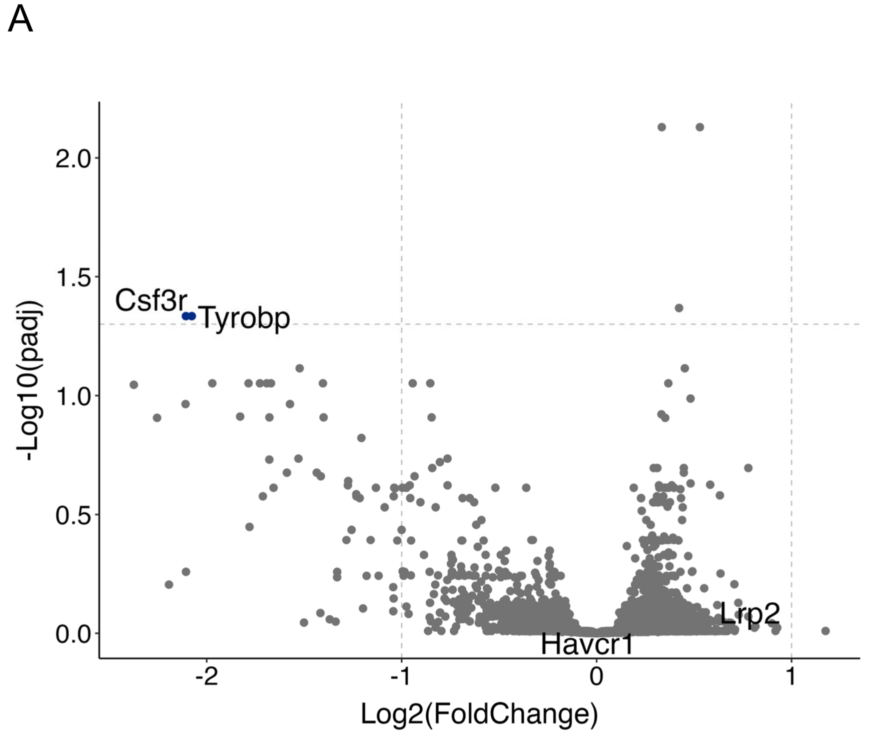

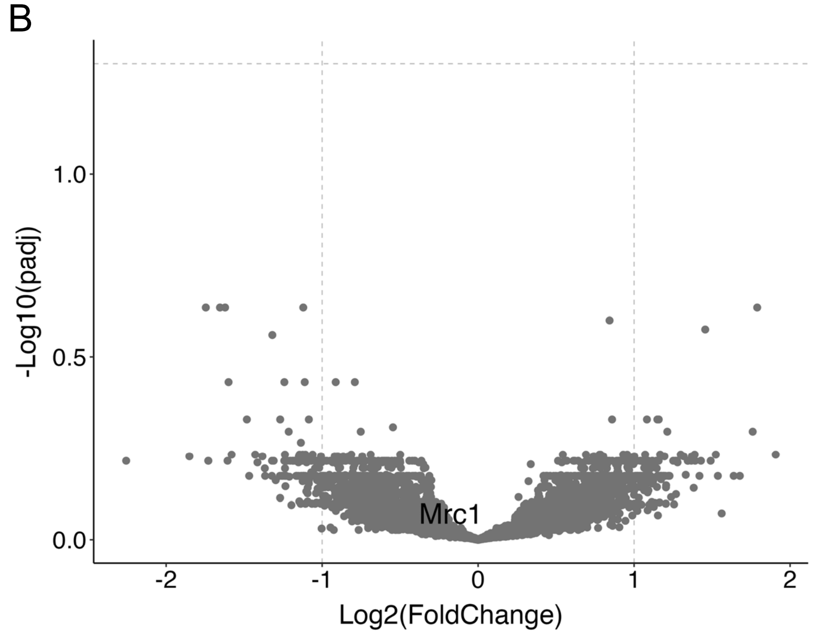

Supplemental Figure 6. Differential gene expression on day 1 post ischemia reperfusion injury (IRI). Two kidney sections/condition were labeled using IBA1 for macrophages and CD10 and PanCK to identify tubules. Regions of Interest (ROIs) in the cortex and the medulla were chosen (2 sections/condition) and cell populations segregated based on their fluorescent label followed by whole transcriptome RNA sequencing. Volcano plots showing differentially expressed genes (padj < 0.05 and abs (log2 Fold Change) > 1) when comparing entospletinib IRI vs vehicle IRI on day 1 from **(A)** tubules (PanCK+CD10+) and **(B)** macrophages (IBA1+).

Supplemental Figure 7

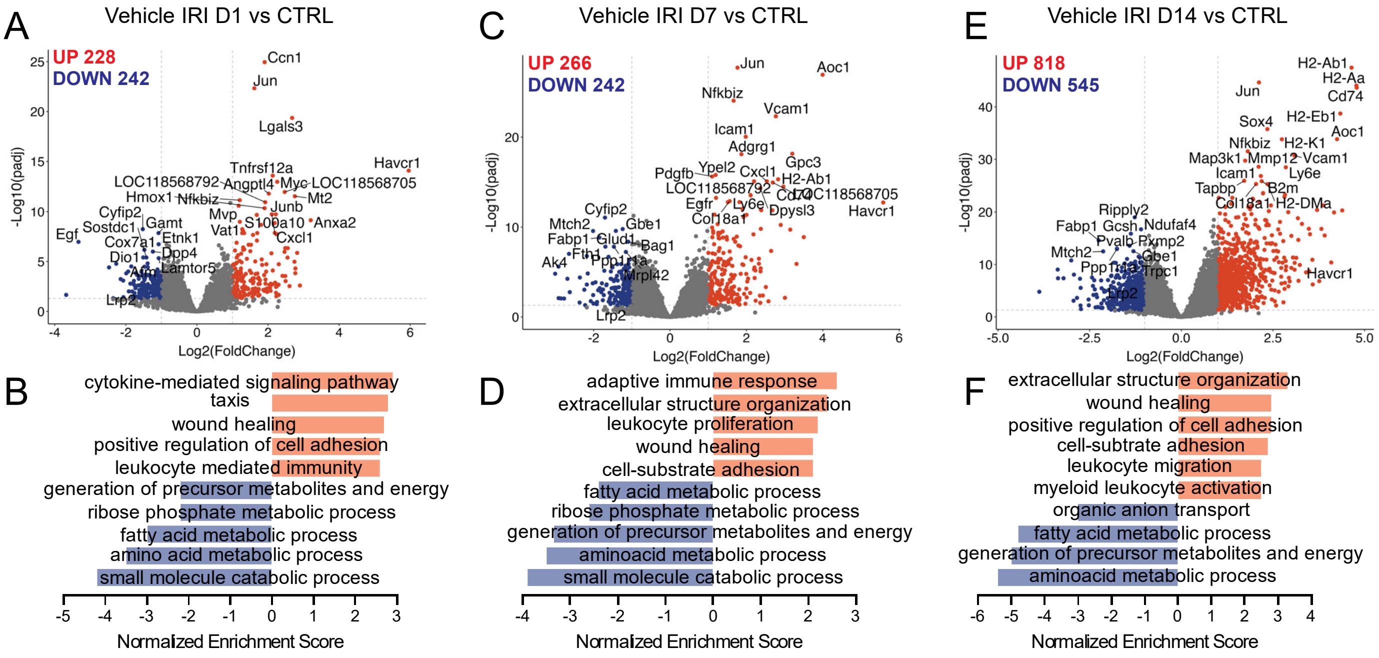

Supplemental Figure 7. Pathway analysis in tubules during AKI-to-CKD transition. Volcano plots derived from digital spatial profiling showing differentially expressed genes (padj < 0.05 and abs (log2 Fold Change) > 1) and Gene Set Enrichment Analysis using Gene Ontology (GO) in tubules (PanCK+ CD10+) from mice undergoing ischemia reperfusion injury (IRI) or uninjured controls. **(A and B)** Vehicle IRI Day 1 vs CTRL, **(C and D)** Vehicle IRI Day 7 vs CTRL, **(E and F)** Vehicle IRI Day 14 vs CTRL.

Supplemental Figure 8

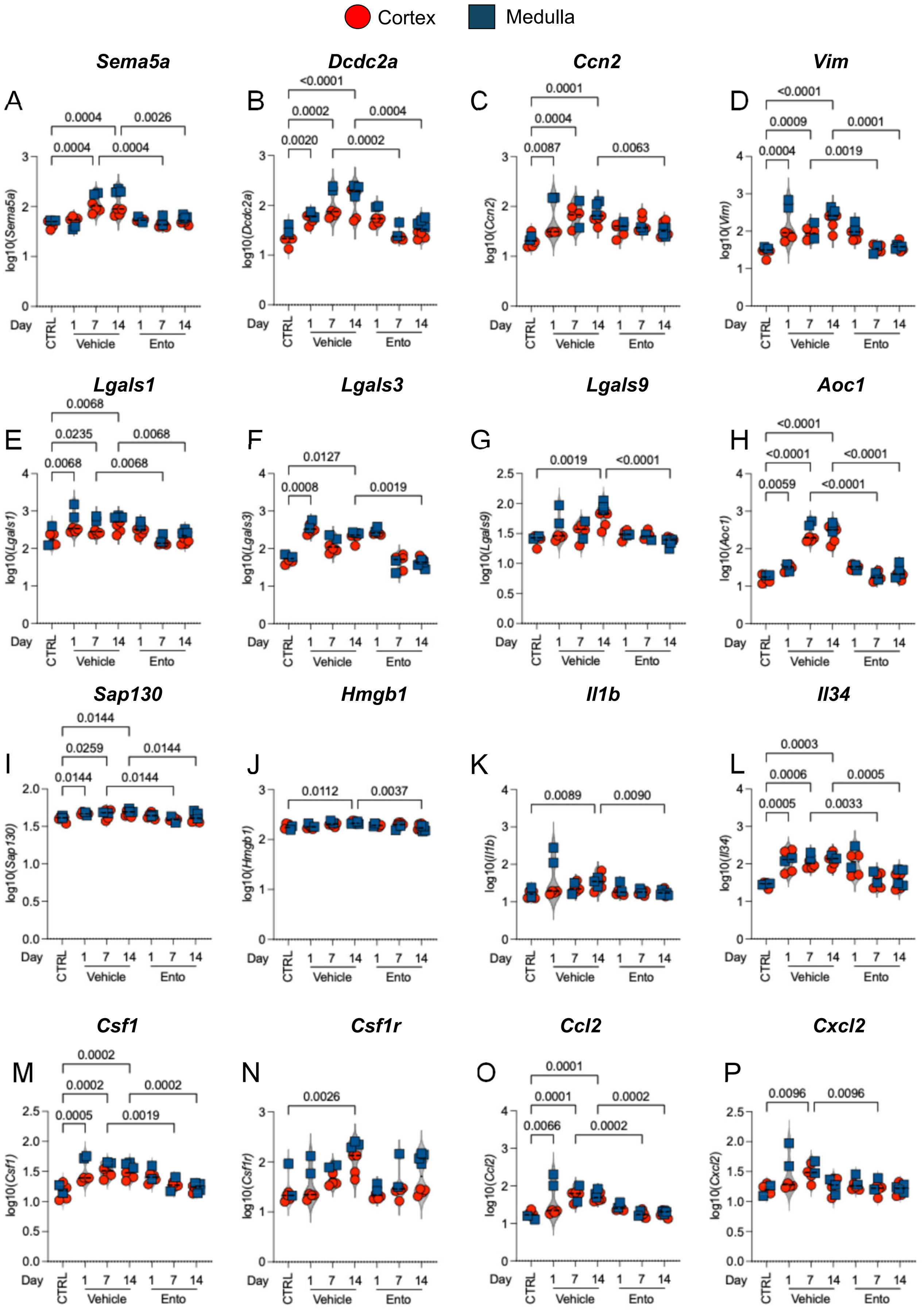

Supplemental Figure 8. Genes associated with injury, inflammation and DAMPs in the tubular compartment. Digital spatial profiling. The log10(normalized gene expression) for key genes associated with injury, inflammation and danger associated molecular patterns (DAMPs) in tubular cells and compared between vehicle and entospletinib (ento)-treated mice over 14 days post ischemia-reperfusion injury (IRI). Differential gene expression for **(A)** *Sema5a*, **(B)** *Dcdc2*, **(C)** *Ccn2*, **(D)** *Vim*, **(E)** *Lgals1*, **(F)** *Lgals3*, **(G)** *Lgals9*, **(H)** *Aoc1*, **(I)** *Sap130*, **(J)** *Hmgb1*, **(K)** *Il1b*, **(L)** *Il34*, **(M)** *Csf1*, **(N)** *Csf1r*, **(O)** *Ccl2*, **(P)** *Cxcl2*. Red circles represent cortical ROIs and blue squares represent ROI's in the medulla. Statistical analysis was performed using Kruskal-Walli's test followed by Dunn's multiple comparison test.

Supplemental Figure 9

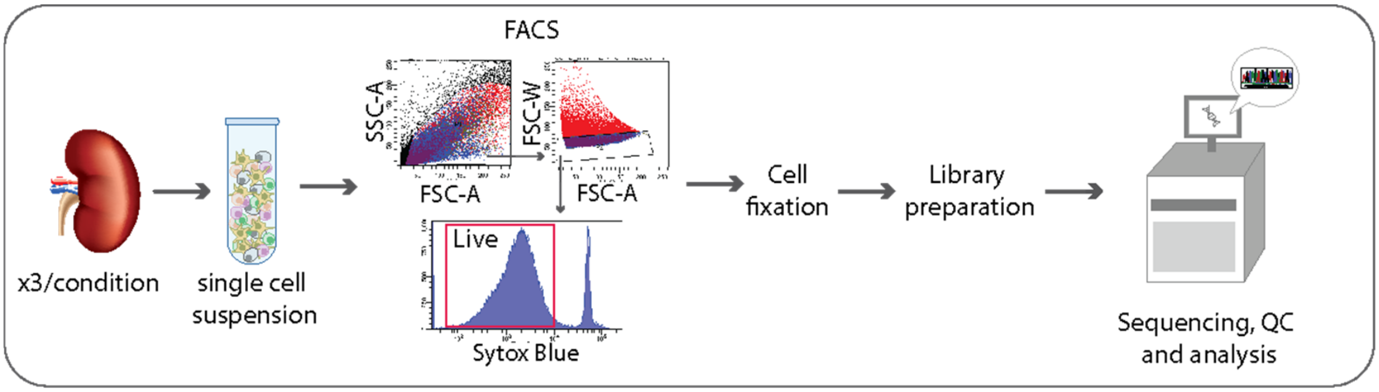

Supplemental Figure 9. Representative workflow of the single-cell RNA-seq experiment enriching for immune cells.

Supplemental Figure 10

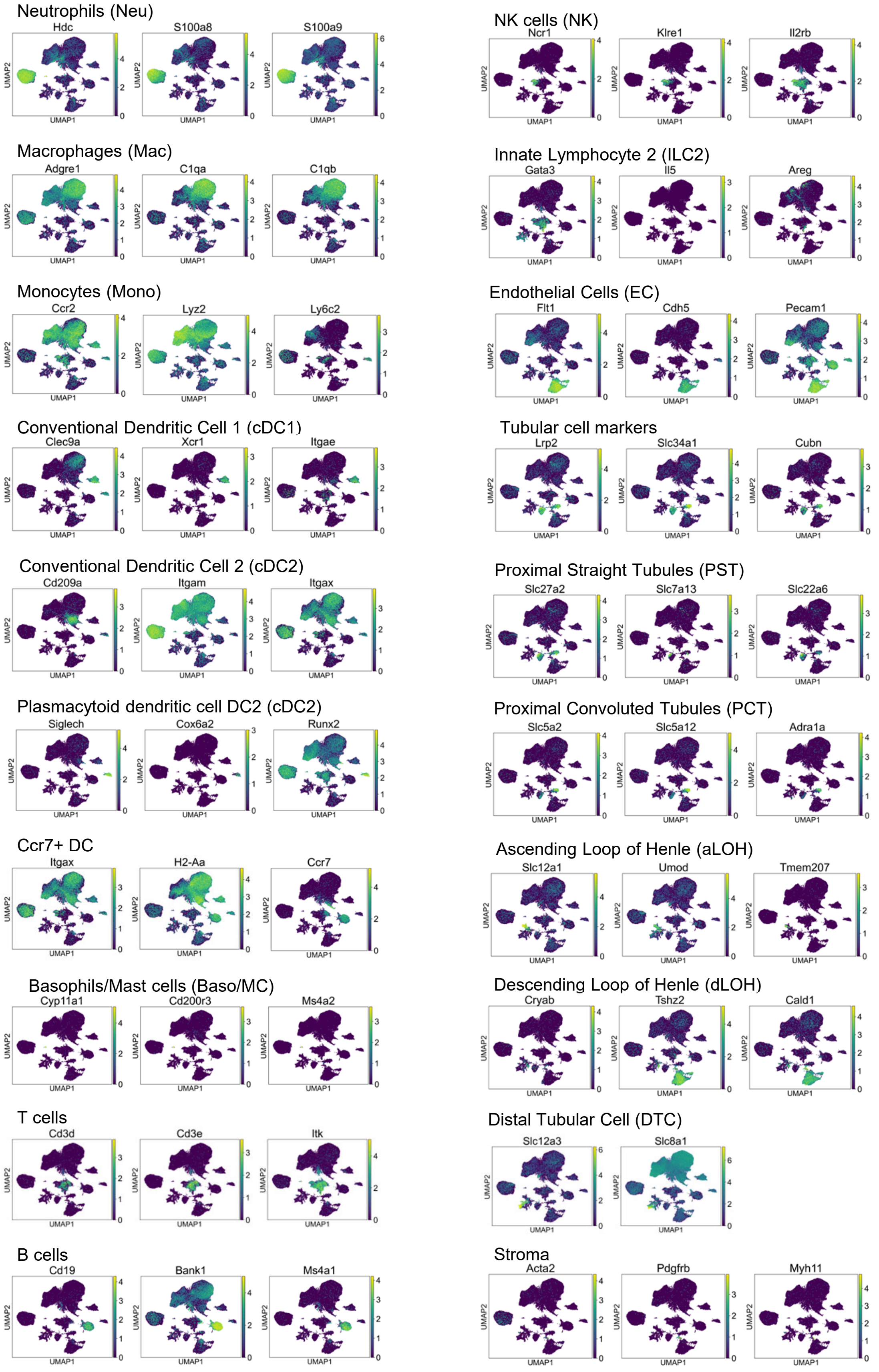

Supplemental Figure 10. Single cell annotation. Density plot illustrating the expression pattern of known genes for each cell annotated.

Supplemental Figure 11

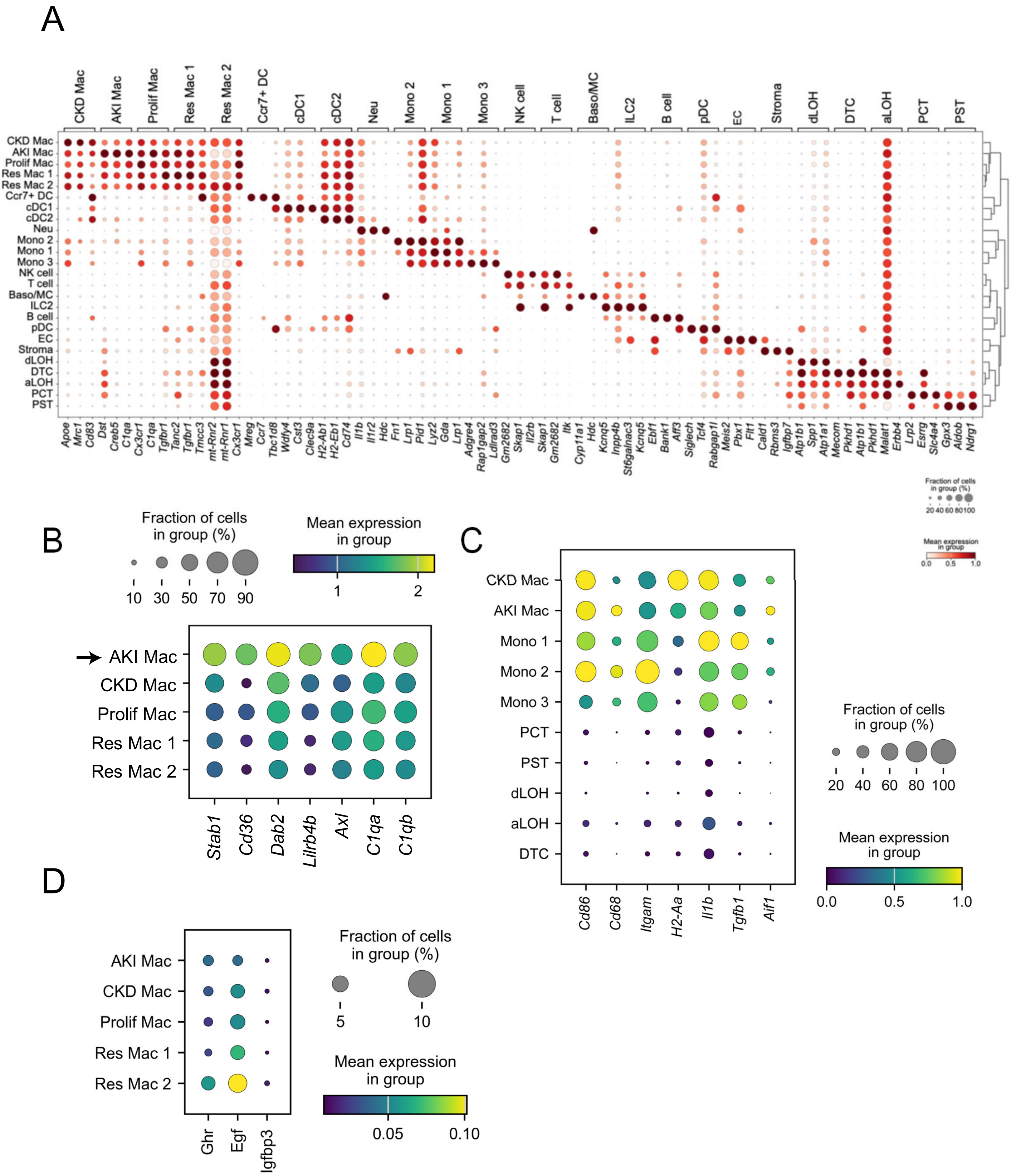

Supplemental Figure 11. Gene expression of annotated cells in scRNA-seq dataset. (A) Dot plot of the 3 top expressed genes in each annotated cell. **(B)** Gene expression in AKI macrophages compared to other macrophage populations. **(C)** Monocyte/macrophage gene expression compared to tubular cells. **(D)** Dot plot showing the expression of *Ghr*, *Egf* and *Igfbp3* in different subsets of macrophages.

Supplemental Figure 12

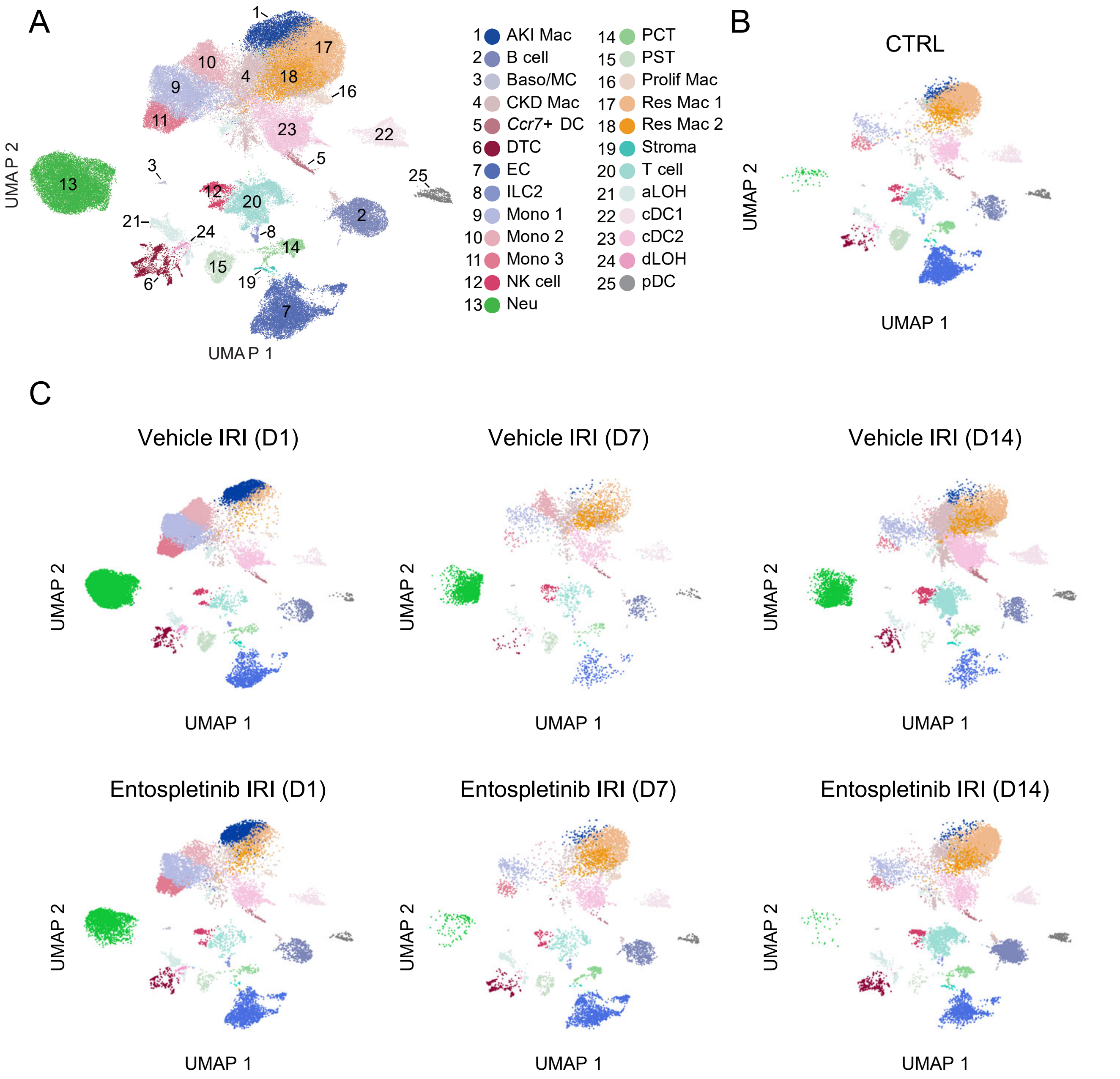

Supplemental Figure 12. Single cell RNA-seq UMAPs for each experimental timepoint and condition *in vivo*. (A) UMAP showing annotated clusters (pooled). **(B, C)** UMAPs showing changes in annotated cluster proportions at baseline and after IRI in vehicle and entospletinib-treated mice.

Supplemental Figure 13

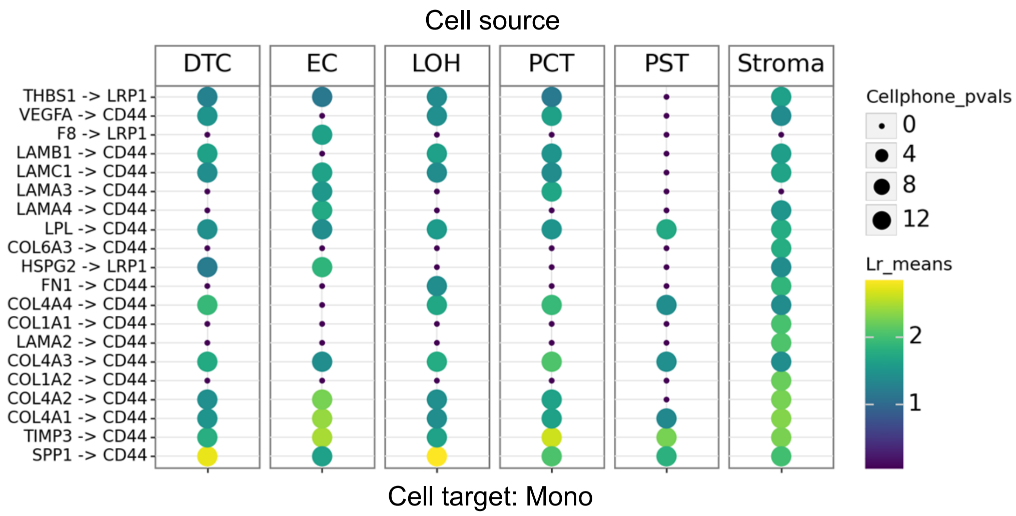

Supplemental Figure 13. Cell-to-cell communication with monocytes. CellPhoneDB analysis of scRNA-seq data showing CD44-mediated interactions between monocytes (target) and source: distal tubular cells (DTC), endothelial cells (EC), ascending and descending loop of Henle cells (LOH), proximal convoluted tubular cells (PCT), proximal straight tubular cells (PST), and stroma.

Supplemental Figure 14

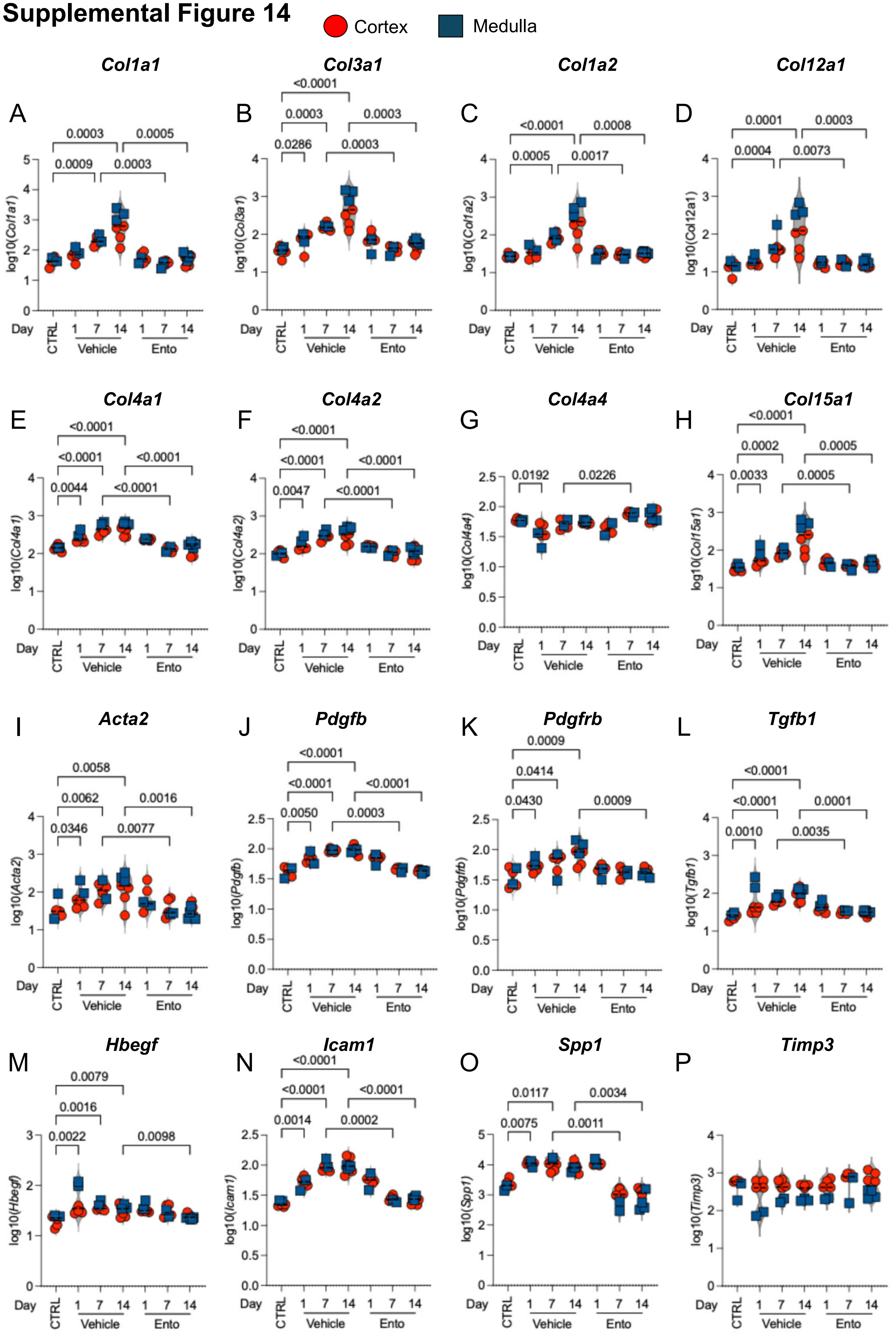

Supplemental Figure 14. Effect of entospletinib in the expression of selected tubular genes. Digital spatial profiling. The log10(normalized gene expression) for selected tubular injury genes and compared between vehicle and entospletinib (ento)-treated mice over 14 days post ischemia-reperfusion injury (IRI). Differential gene expression for **(A)** *Col1a1*, **(B)** *Col3a1*, **(C)** *Col1a2*, **(D)** *Col12a1*, **(E)** *Col4a1*, **(F)** *Col4a2*, **(G)** *Col4a4*, **(H)** *Col15a1*, **(I)** *Acta2*, **(J)** *Pdgfb*, **(K)** *Pdgfrb*, **(L)** *Tgfb1*, **(M)** *Hbegf*, **(N)** *Icam1*, **(O)** *Spp1* and **(P)** *Timp3*. Red circles represent cortical ROIs and blue squares represent ROI's in the medulla. Statistical analysis was performed using Kruskal-Walli's test followed by Dunn's multiple comparison test.

Supplemental Figure 15

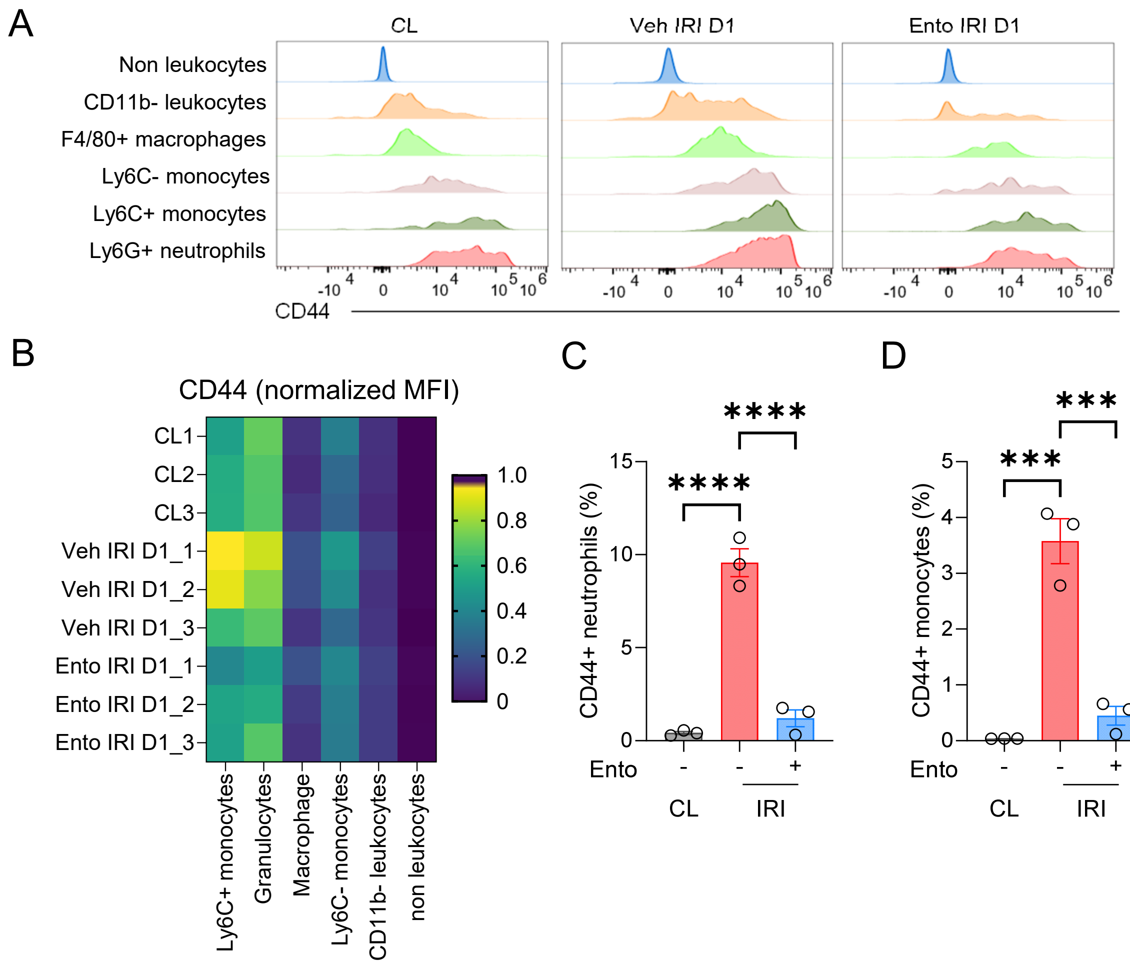

Supplemental Figure 15. CD44 expression in kidney infiltrating leukocytes. C57BL/6 mice received entospletinib or vehicle 30 min before surgery. IRI was induced in the left kidney; the right kidney was used as control. Kidneys were collected on day 1, and CD44 expression was assessed by flow cytometry. **(A)** Representative histograms showing CD44 expression in different leukocytes and non-leukocytes cells for each condition. **(B)** CD44 mean fluorescence intensity was calculated and normalized for each population and condition (n=3). **(C)** Percentage of infiltrating CD44+ neutrophils (n=3) and **(D)** infiltrating CD44+ Ly6C+ monocytes in the kidney (n=3). Statistical analysis was performed using ANOVA followed by Bonferroni's multiple comparisons test (*** p<0.001, **** p<0.0001).

Supplemental Figure 16

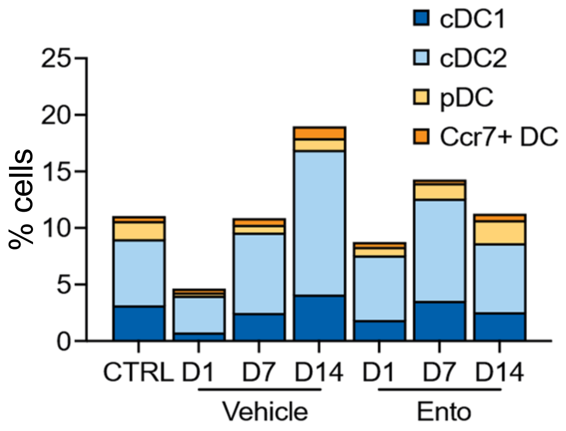

Supplemental Figure 16. Single cell RNA-seq data showing percentage of different dendritic cell subtypes (cDC1, cDC2, pDC and Ccr7+ DC) in the kidney over 14 days post-IRI.

Supplemental Figure 17

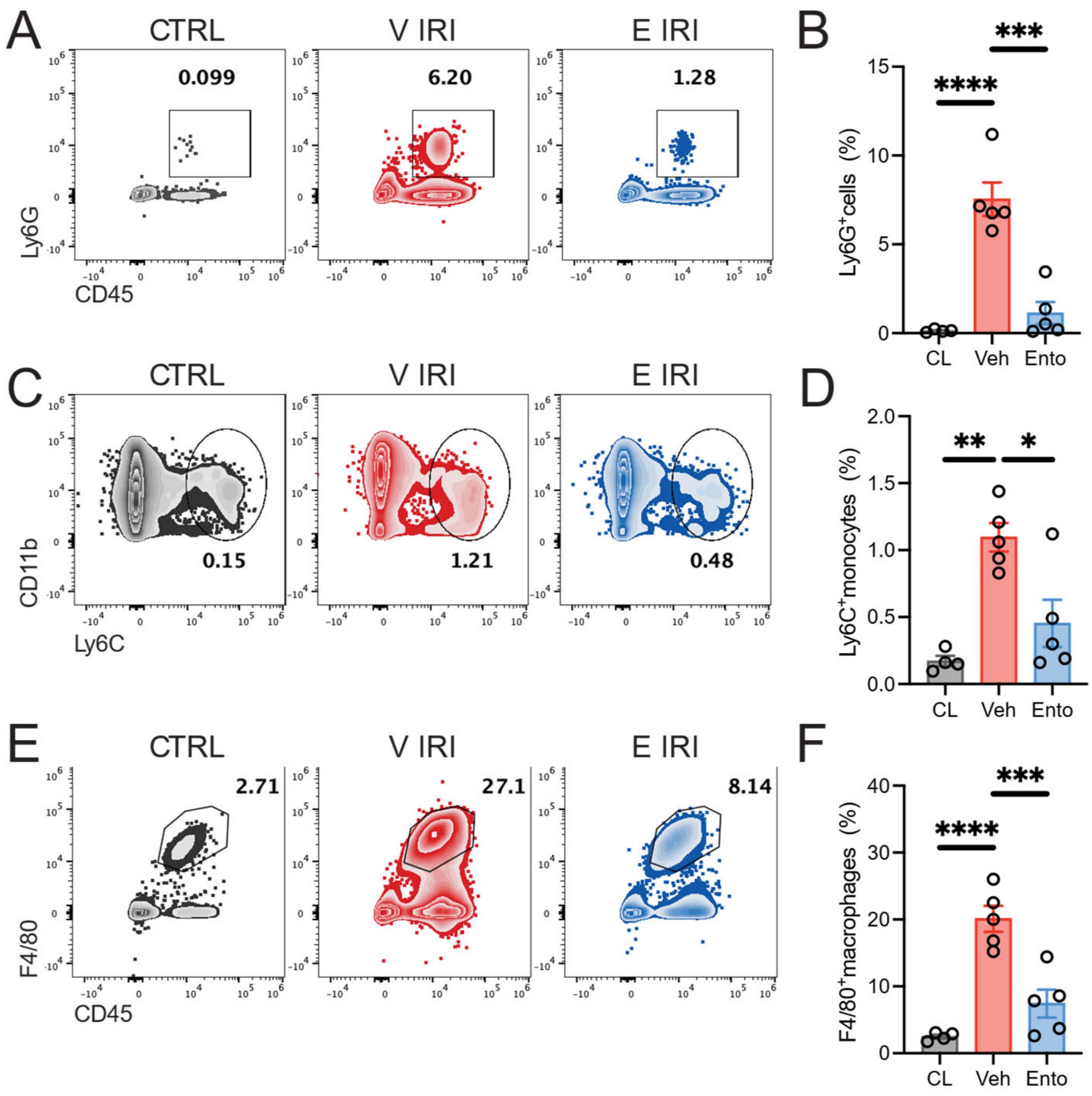

Supplemental Figure 17. Entospletinib modulates leukocyte infiltration post-IRI. IRI was induced in the left pedicle of C57BL/6 mice. One day after surgery, the treatment with entospletinib or vehicle (DMSO) was started. The kidneys were harvested at day 14 (D14) and analyzed by flow cytometry. Representative zebra plots showing the infiltration of **(A)** Ly6G+ neutrophils, **(C)** pro-inflammatory CD11b+Ly6C+ monocytes and **(E)** F4/80+ macrophages in the kidney. The percentage of **(B)** neutrophils, **(D)** monocytes and **(F)** macrophages in the kidney. Data are expressed as mean ± SEM (n=4-5). Statistical analysis was performed by ANOVA followed by Bonferroni's multiple comparisons test (* p<0.05, ** p<0.01, *** p<0.001, **** p<0.0001).

Supplemental Figure 18

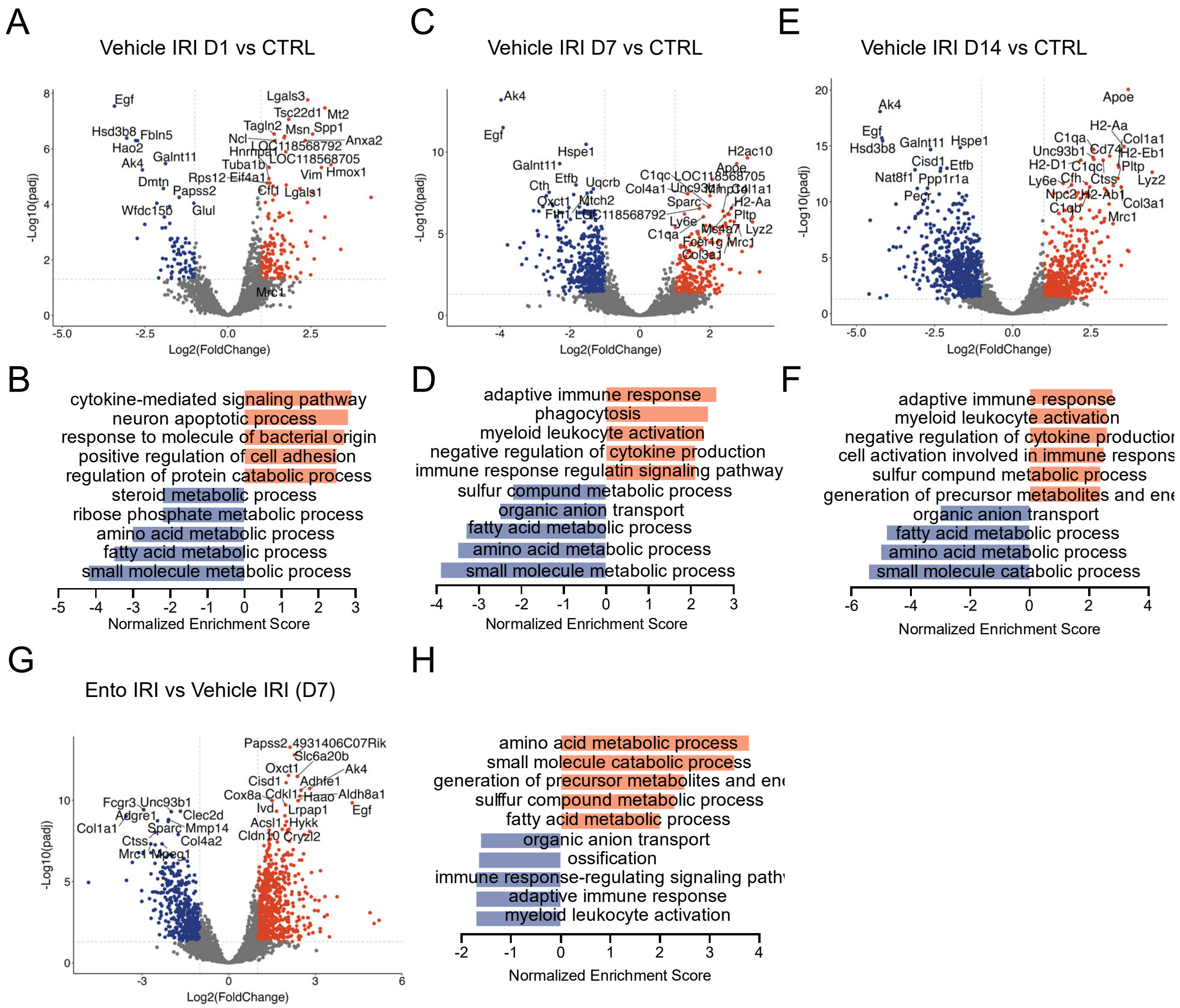

Supplemental Figure 18. Pathway analysis in macrophages during AKI-to-CKD transition. Volcano plots derived from digital spatial profiling showing differentially expressed genes (padj < 0.05 and abs (log2 Fold Change) > 1) and Gene Set Enrichment Analysis using Gene Ontology (GO) in macrophages (IBA1+) from mice undergoing ischemia reperfusion injury (IRI) or uninjured controls (CTRL). **(A and B)** Vehicle IRI Day 1 vs CTRL, **(C and D)** Vehicle IRI Day 7 vs CTRL, **(E and F)** Vehicle IRI Day 14 vs CTRL, entospletinib IRI Day 7 vs vehicle IRI Day 7 (**G** and **H**).

Supplemental Figure 19

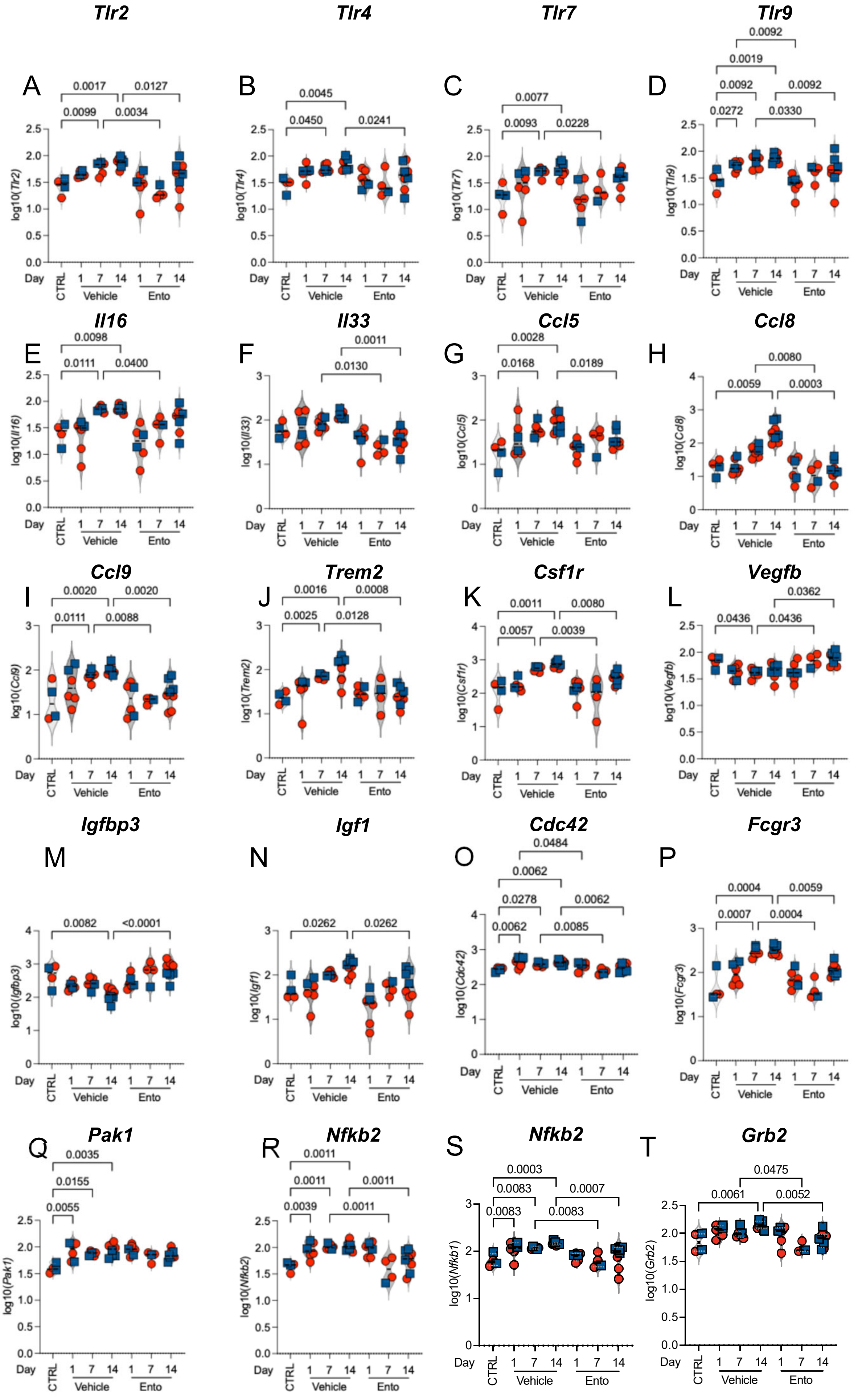

Supplemental Figure 19. Effect of entospletinib in the expression of selected macrophage genes. Digital spatial profiling. The log10(normalized gene expression) for selected genes in IBA1+ macrophages compared between vehicle and entospletinib (ento)-treated mice over 14 days post ischemia-reperfusion injury (IRI). Differential gene expression for **(A)** *Tlr2*, **(B)** *Tlr4*, **(C)** *Tlr7*, **(D)** *Tlr9*, **(E)** *Il16*, **(F)** *Il33*, **(G)** *Ccl5*, **(H)** *Ccl8*, **(I)** *Ccl9*, **(J)** *Trem2*, **(K)** *Csf1r*, **(L)** *Vegfb*, **(M)** *Igfbp3*, **(N)** *Igf1*, **(O)** *Cdc42*, **(P)** *Fcgr3*, **(Q)** *Pak1*, **(R)** *Nfkb2,* **(S)** *Nfkb1* and **(T)** *Grb2***.** Red circles represent cortical ROIs and blue squares represent ROI's in the medulla. Statistical analysis was performed using Kruskal-Walli's test followed by Dunn's multiple comparison test.

Supplemental Figure 20

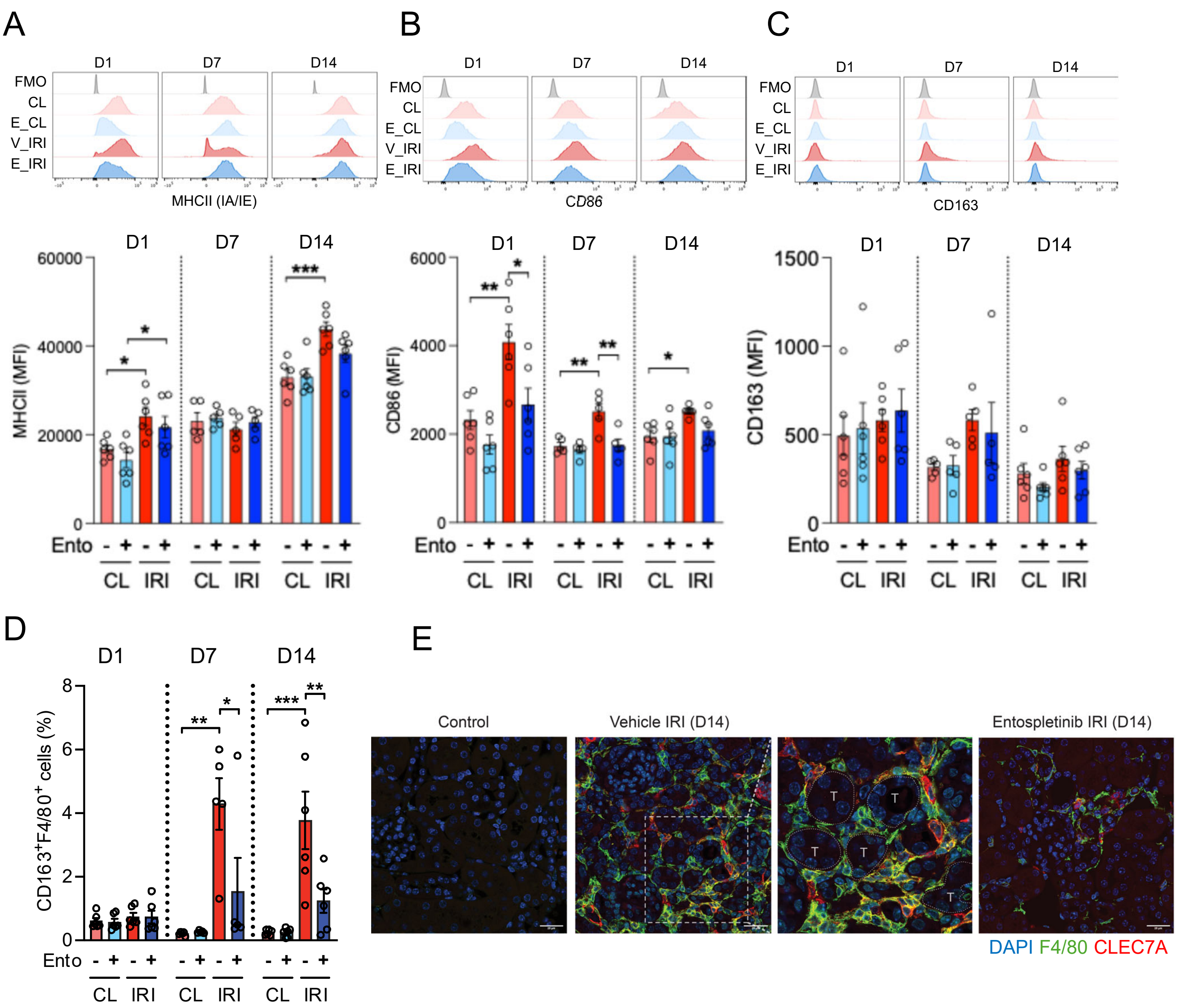

Supplemental Figure 20. Activation markers in macrophages. Flow cytometry of kidney leukocytes probing for **(A)** MHCII **(B)** CD86 and **(C)** CD163 (mean fluorescence intensity, MFI). **(D)** Percentage of CD163+F4/80^{high} cells in kidney leukocytes isolated at day 1, 7 and 14 following ischemia reperfusion injury (IRI). Contralateral (CL) kidneys are used as controls. Statistical analysis was performed by ANOVA followed by Bonferroni's multiple comparisons test (* $p<0.05$, ** $p<0.01$, *** $p<0.001$) (n=4-5). **(E)** Immunofluorescence microscopy probing for CLEC7A and F4/80 in the kidneys of vehicle-treated and entospetinib (ento)-treated mice at 14 days post IRI. Contralateral kidney is used as a negative control.

Supplemental Figure 21

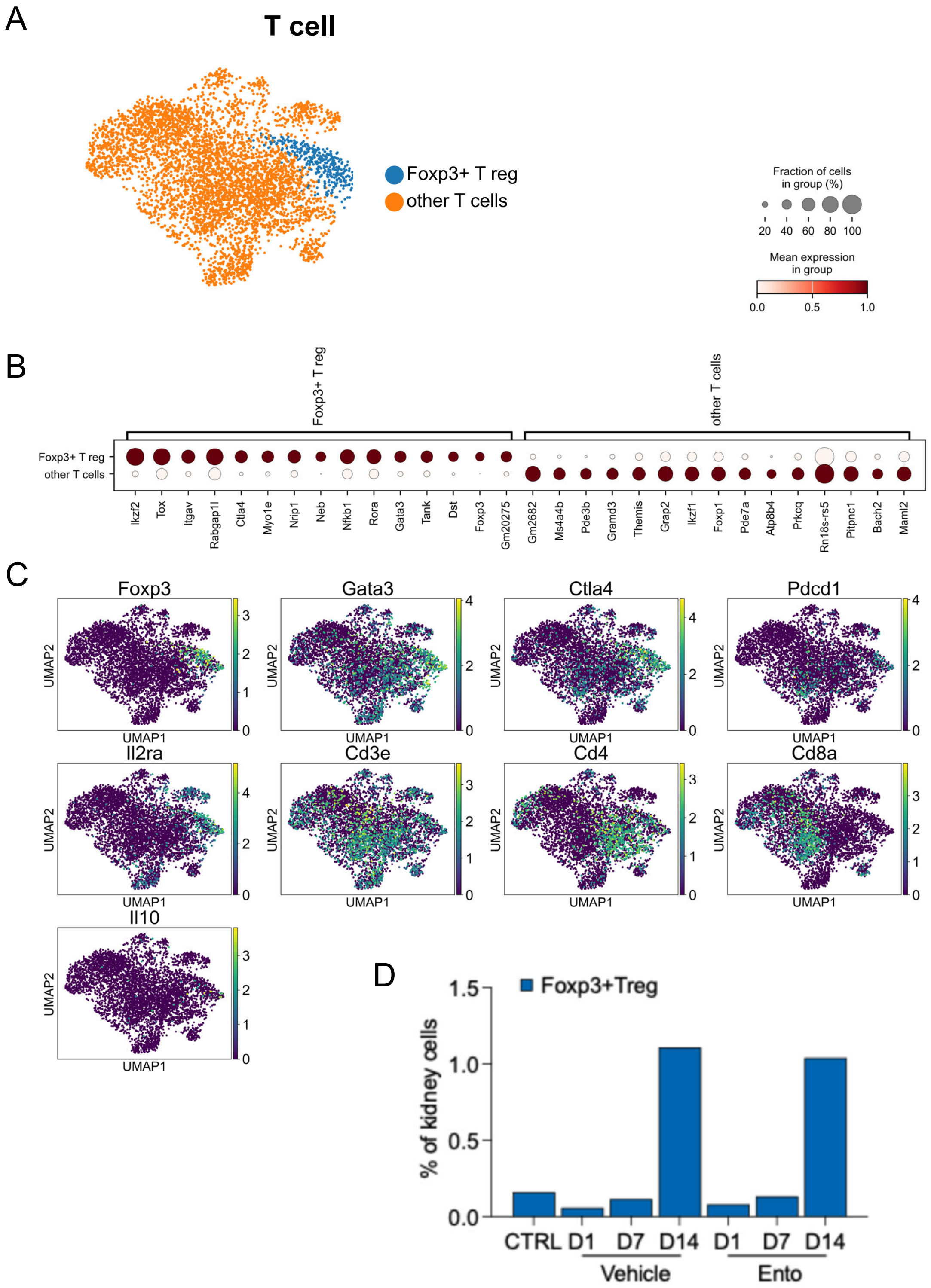

Supplemental Figure 21. *Foxp3*+ Tregs in the kidney. T cell cluster from single cell RNA-seq data was subclustered with a resolution of 0.6 and annotated for regulatory T cells (Treg) based on known markers. **(A)** UMAP of T cells highlighting *Foxp3*+ Treg subcluster. **(B)** Dot plot showing the top 15 expressed genes. **(C)** UMAP for known *Foxp3*+ Treg-expressed genes. **(D)** Percentage of Treg in vehicle and entospletinib (ento)-treated mice over 14 days post ischemia reperfusion injury (IRI).

Supplemental Figure 22

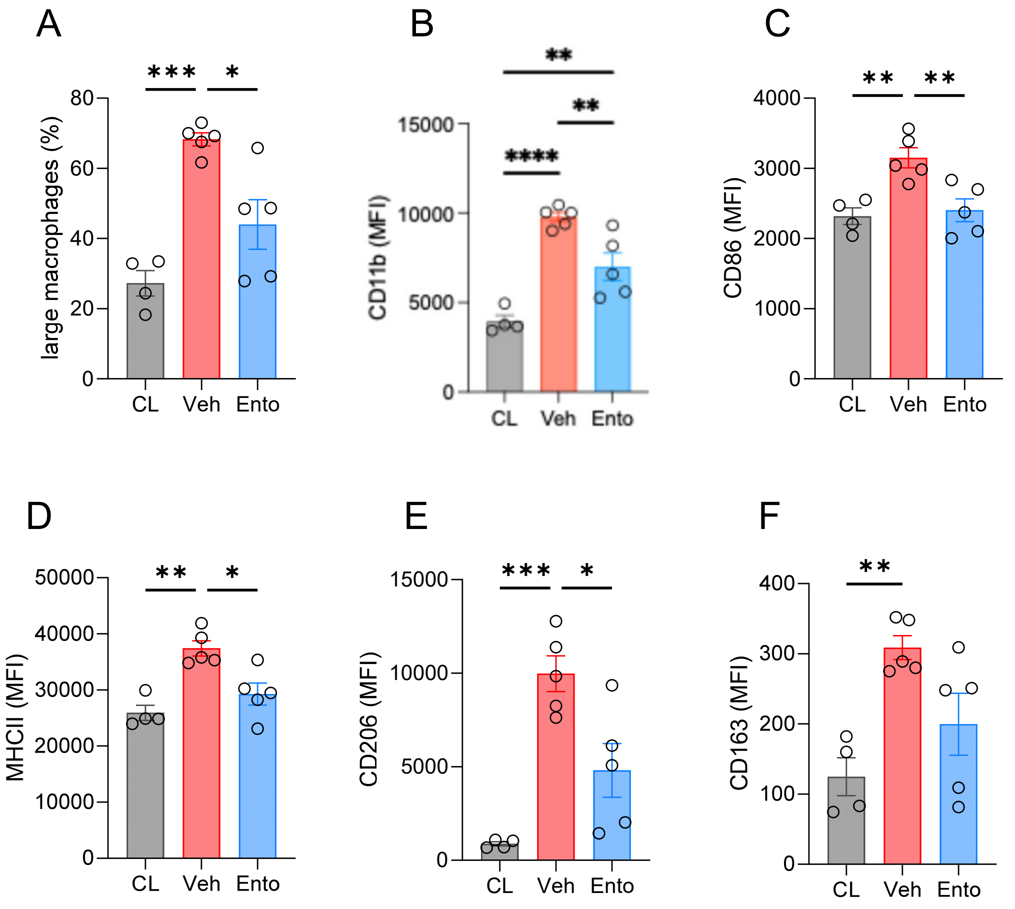

Supplemental Figure 22. Entospletinib attenuates macrophage activation post ischemia reperfusion injury (IRI). IRI was induced in the left pedicle of C57BL/6 mice. One day after surgery, the treatment with entospletinib or vehicle (DMSO) started. The kidneys were harvested at day 14 (D14). The expression of activation markers in F4/80^{high} macrophages was studied by flow cytometry. Percentage of **(A)** large macrophages, **(B)** CD11b, **(C)** CD86, **(D)** MHCII, **(E)** CD206 and **(F)** CD163. Mean fluorescence intensity (MFI), n=4-5. Statistical analysis was performed by ANOVA followed by Bonferroni's multiple comparisons test (* p<0.05, ** p<0.01, *** p<0.001, **** p<0.0001).

Supplemental Figure 23

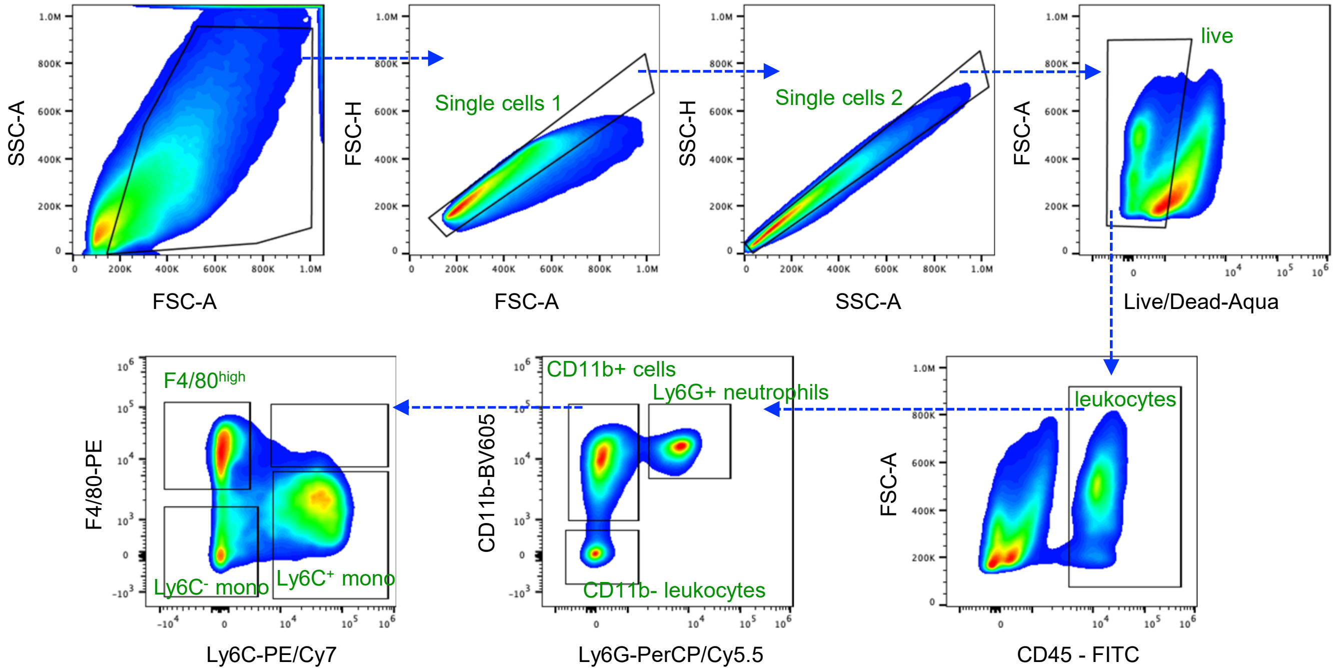

Supplemental Figure 23. Gating strategy to study renal leukocytes by flow cytometry. Renal leukocytes were stained for dead cells, CD45, CD11b, Ly6G, Ly6C and F4/80 and studied by flow cytometry. The figure shows the gating strategy and the phenotype for each immune cell described in this work.

Supplemental Video Legends

Supplemental Video 1. Kidney intravital microscopy in vehicle-treated *LysM*$^{gfp/gfp}$ mice at 24 hours post ischemia reperfusion injury. Neutrophils/monocytes (GFP+ bright green), capillaries (QTRacker, blue), tubules (autofluorescence, dark green). Large GFP+ cells adhere to and crawl along tubules.

Supplemental Video 2. Kidney intravital microscopy in entospletinib-treated *LysM*$^{gfp/gfp}$ mice at 24 hours post ischemia reperfusion injury. Neutrophils/monocytes (GFP+ bright green), capillaries (QTRacker, blue), tubules (autofluorescence, dark green). Few GFP+ cells are present and transiently interact with tubules before returning into circulation.

Supplemental Table 1

KEY REAGENTS

Reagent	catalog #	company	conjugated	dilution/final conc.
Cell Activation Cocktail with Brefeldin A	423303	Biolegend	no	NA
ultra pure LPS	tlr-3pelps	Invivogen	no	1ug/ml
Cyto-Fast™ Fix/Perm Buffer Set	426803	Biolegend	no	NA
Citrate Buffer pH 6.0, 10x, antigen retriever	C9999-1000ML	Sigma-Aldrich	no	1:10
Rabbit Immunoglobulin IgG Fraction	X090302-8	Dako, Agilent	no	1:50
Dako REAL Peroxidase-Blocking Solution	S2023	Dako, Agilent	no	NA
ImmPRESS® HRP Horse Anti-Rabbit IgG Polymer Detection Kit, Peroxidase	MP-7401	Vector	no	NA
ImmPACT® DAB Substrate Kit, Peroxidase (HRP)	SK-4105	Vector	no	NA
Multi Tissue Dissociation Kit 1	130-110-201	Miltenyi	no	protocol
BD Pharm Lyse™ Lysing Solution (10x)	555899	BD Biosciences	no	1:10
Ultra-LEAF™ Purified anti-mouse CD16/32	101330	Biolegend	no	1:100
Zombie Aqua™ Fixable Viability Kit	423101	Biolegend	yes	1:500
SYTOX™ Blue Dead Cell Stain	S34857	Invitrogen™	yes	1:1000
Barcoding Plates	WP100	Parse Bioscience	no	NA
Barcoding Reagents kit	WB100	Parse Bioscience	no	NA
cDNA Amplification reagents kit	WC100	Parse Bioscience	no	NA
Fragmentation reagents kit	WX200	Parse Bioscience	no	NA
Library preparation accessories	WA100	Parse Bioscience	no	NA
4°C Library Preparation Reagents	WA200	Parse Bioscience	no	NA
Evercode cell fixation v2 kit	WF300	Parse Bioscience	no	NA
entospletinib (GS-9973)(a SYK inhibitor)	206107	MedKoo	no	20mg/kg/0.1-1uM
Propidium Iodide	P1304MP	ThermoFisher	no	2ug/ml

KEY ANTIBODIES

antibody	catalog	company	conjugated	dil	clone
CD45	103105	Biolegend	PE	1:200	30-F11
CD45	103107	Biolegend	FITC	1:200	30-F11
CD11b	101257	Biolegend	BV605	1:100	M1/70
CD11b	101208	Biolegend	PE	1:100	M1/70
CD11c	117323	Biolegend	APC/Cy7	1:100	N418
CD86	105027	Biolegend	PerCP/Cy5.5	1:100	GL-1
CD86	105007	Biolegend	PE	1:100	GL-1
CD86	105019	Biolegend	AF647	1:200	GL-1
F4/80	123110	Biolegend	PE	1:100	BM8
F4/80	123132	Biolegend	BV421	1:100	BM8
F4/80	565410	BD	PE	1:100	T45-2342
IA/IE	107607	Biolegend	PE	1:200	M5/114.15.2
IA/IE	107606	Biolegend	FITC	1:200	M5/114.15.2
Ly6C	128018	Biolegend	PE/Cy7	1:200	HK1.4
Ly6G	127653	Biolegend	PerCP/Cy5.5	1:100	1A8
CD206	141711	Biolegend	AF647	1:100	C068C2
CD163	155319	Biolegend	PE/Cy7	1:100	S15049I
SYK	646003	Biolegend	PE	1:100	5F5
CD31	102423	Biolegend	BV421	1:100	390
CD44	103018	Biolegend	AF647	1:100	IM7
LTL	FL-1321	Vector	FITC	1:200	NA
LEL	DL-1174	Vector	DyLight488	1:200	NA
Dectin-1/ Clec7a (E3P5W) Rabbit mAb #30260	30260T	NEB	unconjugated	1:400	E3P5W
Anti-Collagen I + Collagen III antibody	ab34710	Abcam	unconjugated	1:200	Polyclonal
Galectin 9	ab69630	Abcam	unconjugated	1:300	Polyclonal
Vimentin (D21H3) XP® Rabbit mAb #5741	5741T	NEB	unconjugated	1:400	D21H3
Phospho-Syk (Tyr525/526) (C87C1) Rabbit mAb	2710S	CST	unconjugated	1:1000	C87C1
Syk (D3Z1E) XP® Rabbit mAb	13198S	CST	unconjugated	1:1000	D3Z1E
GAPDH (Rb)	2118S	CST	unconjugated	1:1000	14C10
alpha-SMA	ab7817	Abcam	unconjugated	1:200	1A4
F4/80	ab6640	Abcam	unconjugated	1:200	CI:A3-1
Goat anti-Rabbit HRP secondary	111-035-003	Jackson Immuno	unconjugated	1:5000	Polyclonal
CD206	ab64693	Abcam	unconjugated	1:200	Polyclonal
CD206	AF2535-SP	R&D	unconjugated	1:100	Polyclonal
CD45	550539	BD Pharmigen	unconjugated	0.07638889	30-F11
Gt anti-Rat AF488	A11006	Invitrogen	AF488	1:300	Polyclonal
Gt anti-Ms AF568	A11004	Invitrogen	AF568	1:300	Polyclonal
Gt anti-Rb AF647	A27040	Invitrogen	AF647	1:300	Polyclonal
DK anti-Gt AF568	A11057	Invitrogen	AF568	1:300	Polyclonal
DK anti-Rb AF647	A31573	Invitrogen	AF647	1:300	Polyclonal
Prolong Gold antifade with DAPI	P36931	Invitrogen	DAPI	1:300	Polyclonal

KEY EQUIPTMENT

equipment	source	identfier
Attune NxT Flow Cytometer (14 colors)	Thermofisher	https://www.thermofisher.com/
Leica SP8 confocal microscope	Leica	https://www.leica-microsystems.com/
Aperio	Leica	https://www.leicabiosystems.com/
BD FACSAria III	BD Biosciences	https://www.bdbiosciences.com/

KEY SOFTWARE

SOFTWARE	source	identfier
FlowJo v10.10	FlowJo	https://www.flowjo.com/
LAS X Life Science	Leica	https://www.leica-microsystems.com/
GraphPad Prism v10	GraphPad/Dotmatics	https://www.graphpad.com/
EndNote v21.2	EndNote	https://endnote.com/
Python 3.1	Python	Welcome to Python.org
R 4.4.0	R	R: The R Project for Statistical Computing (r-project.org)
Rstudio 2024.04.1	Posit	RStudio Desktop - Posit
Scanpy	Scanpy	Scanpy – Single-Cell Analysis in Python — scanpy
CellChat	CellChat	jinworks/CellChat: R toolkit for inference, visualization and analysis of cell-cell communication from single-cell and spatially resolved transcriptomics (github.com)
DESeq2	Bioconductor	Bioconductor - DESeq2
Parse_pipeline v1.1	Parse Bioscience	https://www.parsebiosciences.com/
ShinyGO 0.80	South Dakota State University	http://bioinformatics.sdstate.edu/go/

Supplemental Table 2

QC paramters used in this report			
**Step**	**Filter name**	**Threshold**	**Description**
SegmentQC	minSegmentReads	1000	Minimum number of reads
	percentTrimmed	80	Minimum % of reads trimmed
	percentStitched	80	Minimum % of reads stitched
	percentAligned	75	Minimum % of reads aligned
	percentSaturation	50	Minimum sequencing saturation (%)
	minNuclei	20	Minimum number of nuclei estimated
	minArea	1000	Minimum segment area
ProbeQC	minProbeRatio	0.1	Geometric mean of a given probe / geometric mean of all probe
	percentFailGrubbs	20	An outlier according to the Grubb's test (%)
LOQ	loqCutoff	2	LOQ cut off value
	loqMin	2	LOQ minimum value
GeneQC	geneDetectionRateThre	0.05	Minimum gene detection rate
