# Supplementary figures and images for: The spleen tyrosine kinase inhibitor entospletinib resolves inflammation to promote repair following acute kidney injury
Source: JCI Insight. 2025 Aug 22;10(16):e189601. doi: 10.1172/jci.insight.189601 (PMC12406726; doi:10.1172/jci.insight.189601)

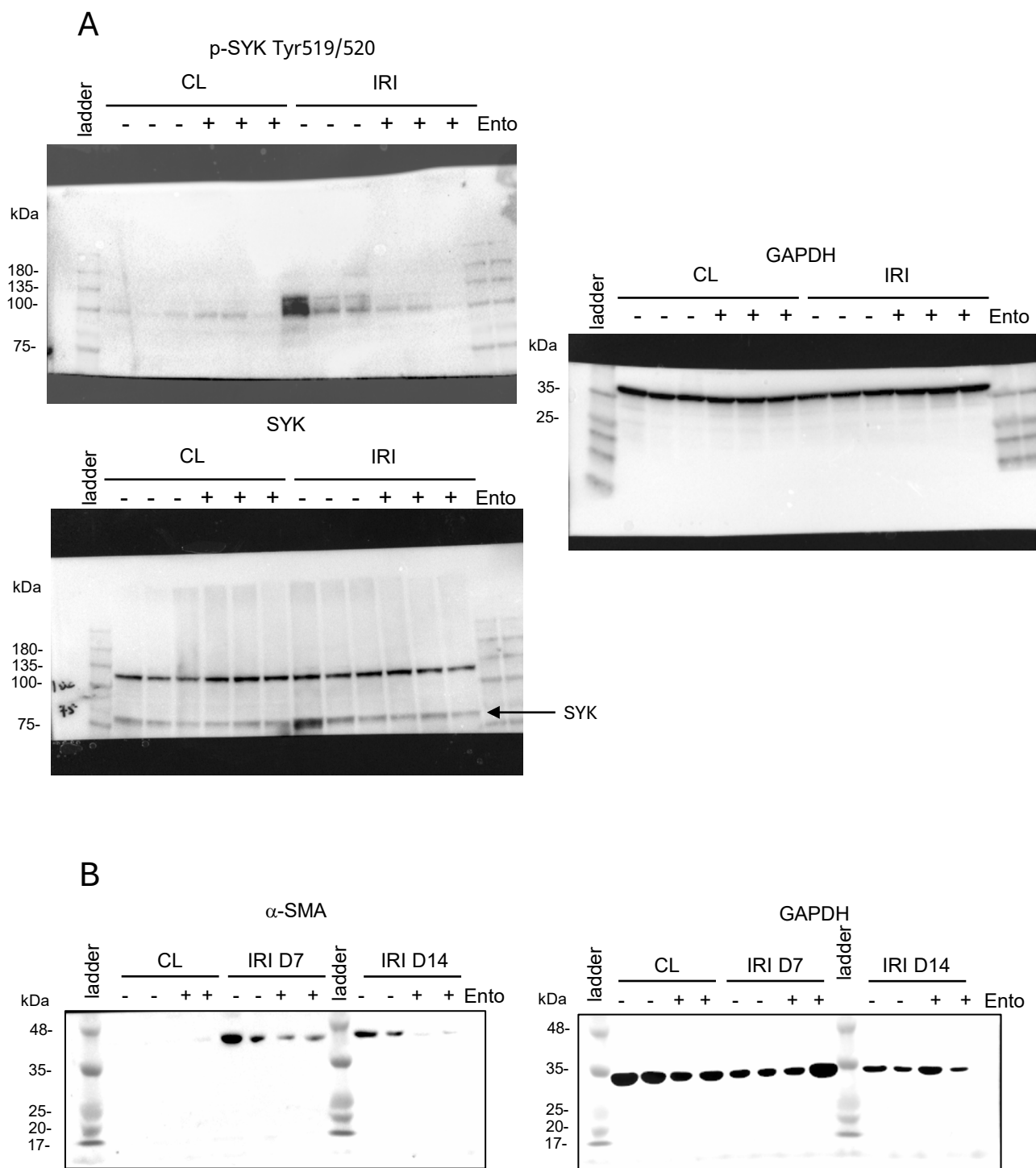

Uncropped immunoblots presented in (A) figure 1 and (B) figure 2.

Supplement: Unedited blot and gel images [file jciinsight-10-189601-s017.pdf]
